# Structural basis for anaerobic alkane activation by a multisubunit glycyl radical enzyme

**DOI:** 10.1073/pnas.2510389122

**Published:** 2025-08-04

**Authors:** Mary C. Andorfer, Talya S. Levitz, Jian Liu, Ankush Chakraborty, Devin T. King-Roberts, Delight Nweneka, Christa N. Imrich, Catherine L. Drennan

**Affiliations:** ^a^Department of Chemistry, Michigan State University, East Lansing, MI 48824; ^b^Department of Biology, Massachusetts Institute of Technology, Cambridge, MA 02139; ^c^HHMI, Massachusetts Institute of Technology, Cambridge, MA 02139; ^d^Department of Biological Engineering, Massachusetts Institute of Technology, Cambridge, MA 02139; ^e^Department of Chemistry, Massachusetts Institute of Technology, Cambridge, MA 02139

**Keywords:** C–H functionalization, anaerobic hydrocarbon degradation, environmental remediation, X-succinate synthase, fumarate addition

## Abstract

X-succinate synthases (XSSs) are glycyl radical enzymes (GREs) that catalyze the addition of hydrocarbons to fumarate via radical chemistry, thereby activating them for microbial metabolism. To date, the only structurally characterized XSS is benzylsuccinate synthase (BSS), which functionalizes toluene. A distinct subclass of XSSs acts on saturated hydrocarbons, which possess much stronger C(sp^3^)–H bonds than toluene, suggesting mechanistic and structural differences from BSS. Here, we use cryogenic electron microscopy to determine the structure of one such enzyme, (1-methylalkyl)succinate synthase (MASS) from *Azoarcus* strain HxN1, which functionalizes *n*-alkanes (C6–C8). The structure reveals an asymmetric dimer in which both sides contain a catalytic α-subunit and accessory γ-subunit. One α-subunit also binds two additional subunits, β and δ. The β-subunit binds a [4Fe–4S] cluster and adopts a fold similar to BSSβ. The β-subunit appears to regulate the flexibility of the α-subunit to enable opening of the active site, affording the binding of *n*-alkane substrates. The δ-subunit, which lacks homology to known GRE subunits, adopts a rubredoxin-like fold that binds a single Fe ion, an architecture not previously reported for GREs. MASSδ occupies the same region of the α-subunit as the activating enzyme (AE) and may regulate the conformational changes required for glycyl radical installation. Structural comparisons between MASS and BSS reveal differences in how fumarate is bound and show amino acid substitutions that could account for the binding of alkanes versus toluene. Together, this structure offers insight into anaerobic alkane activation via fumarate addition.

## Significance

Glycyl radical enzymes (GREs) enable anaerobic microbes to break down components of crude oil in environments that are resistant to traditional remediation methods. These enzymes can activate saturated alkanes, which constitute a major fraction of crude oil but are chemically inert due to their strong C–H bonds. Unlike aerobic enzymes that require molecular oxygen to initiate alkane oxidation, GREs function in the absence of oxygen and catalyze C–C bond formation. Here, we present the structure of a GRE that reveals how its accessory subunits coordinate radical generation and capture *n*-alkane substrates for activation. This work provides insight into how nature performs challenging chemistry in oxygen-free environments, with implications for bioremediation and sustainable biocatalysis.

**Fig. 1.**
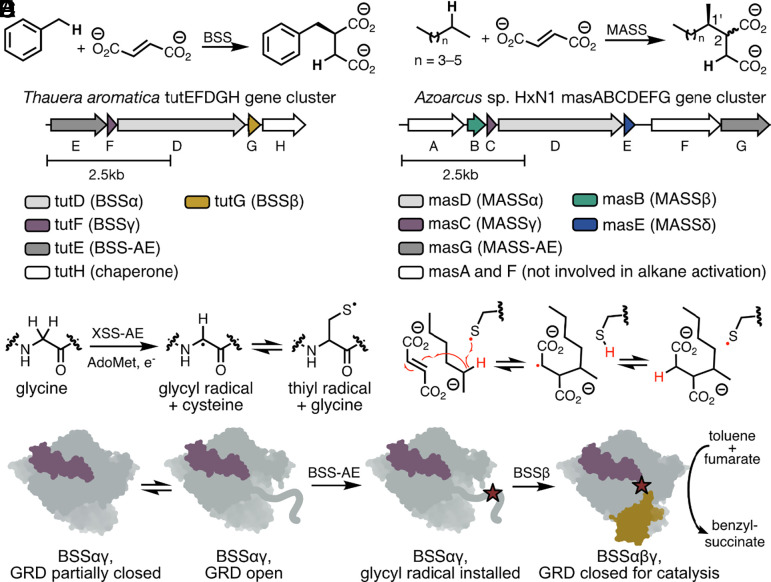
Hydroalkylation of fumarate by XSSs. (*A*) Benzylsuccinate synthase (BSS) catalyzes the addition of toluene to fumarate and is encoded by the tutEFDGH gene cluster within *Thauera aromatica* (GenBank: AF113168.1). (*B*) (1-methylalkyl)succinate synthase (MASS) catalyzes the addition of medium chain *n*-alkanes to fumarate and is encoded by the mas gene cluster (GenBank: AM748709.1). (*C*) An activating enzyme (AE) converts a glycine within the XSS to a glycyl radical. This Gly radical can form a transient thiyl radical on a nearby cysteine residue. (*D*) The thiyl radical abstracts an H atom from the hydrocarbon substrate, and the resulting hydrocarbyl radical is added to fumarate. The succinyl radical reabstracts an H atom from Cys to regenerate the thiyl radical. (*E*) The BSSαγ complex can adopt a conformation that enables the glycyl radical domain (GRD) of BSSα to bind within the active site of the AE. After the glycyl radical (denoted by red star) is installed by the AE, BSSβ binds to close the GRD within the active site of BSSα and to plug the substrate channel. The BSSαβγ complex is required for hydroalkylation.

Hydrocarbons are abundant throughout the environment, and the high bond dissociation energies of C–H bonds within hydrocarbons make them minimally reactive. Crude oil is a complicated mixture of inert hydrocarbons, making clean-up of crude oil spills a challenge. In nature, microbes commonly use oxygenases to activate hydrocarbons for aerobic degradation; however, hydrocarbons are also abundant in anaerobic environments, including oil deposits and anoxic sediments. Although anaerobic transformations often proceed more slowly than their aerobic counterparts, they occur across vast ecological niches and make substantial contributions to the global carbon cycle (1). Without the presence of O_2_, some microbes have developed other enzymatic pathways to activate hydrocarbons. Hydrocarbon addition to fumarate is thought to be the most prevalent of these pathways, although hydroxylation and carboxylation have also been proposed (2). A range of hydrocarbons can be activated through fumarate addition, which includes aromatic hydrocarbons [e.g., toluene and 2-methylnaphthalene, Fig. 1*A* (3, 4)] and saturated hydrocarbons [e.g., *n*-alkanes, Fig. 1*B* (5, 6)] (2). Fumarate addition is accomplished by X-succinate synthases (XSSs), which are members of the glycyl radical enzyme (GRE) superfamily (7, 8). As members of the GRE superfamily, XSSs harbor a glycyl radical within the active site of the enzyme. The radical is installed by a radical S-adenosylmethionine (SAM, AdoMet) activating enzyme (AE), which uses AdoMet, an iron-sulfur cluster, and an electron to abstract a hydrogen atom from the essential glycine residue (Fig. 1*C*). The glycyl radical can abstract an H atom from a proximal cysteine residue to form a transient thiyl radical, which is thought to initiate GRE catalysis through H-atom abstraction from substrate (Fig. 1*C*).

The most well-characterized of the XSSs is benzylsuccinate synthase (BSS). BSS isoenzymes from *Thauera aromatica* (9) and *Azoarcus* strain T (10) were first characterized in the 1990s. BSS catalyzes the stereoselective conversion of toluene and fumarate to *R*-benzylsuccinate (Fig. 1*A*) (10–12). The thiyl radical within BSS abstracts an H-atom from the methyl group of toluene, resulting in a benzylic radical that adds to fumarate. The benzylsuccinyl radical reabstracts an H-atom from cysteine to regenerate the glycyl radical. A syn-addition of the benzylic radical and reabstracted H-atom to fumarate has been shown to occur (13, 14). The initial C–H bond cleavage is thought to be involved in the rate-limiting step, as a large kinetic isotope effect is observed using deuterated toluene (14, 15). The bond dissociation energy (BDE) for a benzylic C–H bond is much lower than that of an aliphatic C–H bond; however, many XSSs that functionalize alkanes (alkyl-SSs) are also known to exist (2, 16–19). The denitrifying betaproteobacterium *Azoarcus* sp. strain HxN1 has been shown to degrade saturated alkanes, including *n*-hexane, *n*-heptane, and *n*-octane (16, 20, 21). A homolog of the catalytic BSS subunit has previously been identified in strain HxN1 and is thought to catalyze the addition of fumarate to these alkanes at the C2 position, thus producing (1-methylalkyl)succinates (5). As such, this homolog is referred to as (1-methylalkyl)succinate synthase (MASS). No structural data for an alkyl-SS are currently available. Here, we set out to structurally characterize the MASS from strain HxN1.

In addition to initial classification of MASS from strain HxN1 as a GRE that functionalizes alkanes through fumarate addition, the stereochemistry of this MASS homolog has previously been investigated. When strain HxN1 is anaerobically grown with *n*-hexane, two diastereomers of (1-methylpentyl)succinate are formed [(2*R*,1′*R*)- and (2*S*,1′*R*)-(1-methylpentyl)succinate, Fig. 1*B*] (16, 22). This finding could suggest that hydroalkylation within MASS may not be stereoselective; however, the high stereoselectivity observed in BSS raises the possibility that fumarate addition in MASS is selective, and that the alternate diastereomer arises from the activity of an unknown epimerase (1, 22, 23). Further stereochemical investigations of (1-methylpentyl)succinate formation using deuterated substrates demonstrated that the C–H activation of alkane is stereospecific, and the addition to fumarate results in an inversion of configuration at C2 of the alkane (22). This inversion is consistent with the transformation occurring through a concerted mechanism (Fig. 1*D*), which could explain how such a high difference in BDE between the S–H of cysteine and C–H of alkane could be overcome. Currently, all studies of MASS have been performed within a cellular context, and even more can be gleaned if purified enzyme could be used in biochemical studies (24).

All characterized GREs house their active site within a 10-stranded β/α barrel (8). Centered in the barrel are two loops; one contains the glycyl radical species (Gly loop) and the other contains the catalytic cysteine (Cys loop). These loops are often buried, requiring a conformational change of the glycyl radical domain (GRD) to expose the Gly loop for activation and a substrate channel to deliver the substrate to the Cys loop. To date, there are two structurally characterized GREs that contain accessory subunits in addition to the large catalytic subunit: 4-hydroxyphenylacetate decarboxylase (HPAD) (25) and BSS (26, 27). HPAD contains a small (9.5 kDa) subunit on the surface of the large subunit. The smaller subunit, the function of which is still unknown, binds two [4Fe–4S] clusters and is important for soluble expression of the large subunit when heterologously expressed in *Escherichia coli* (28). BSSα, the catalytic subunit of BSS, is known to bind 2 accessory subunits – BSSγ and BSSβ (26, 29). BSSγ contains a [4Fe–4S] cluster and, like HPAD’s accessory subunit, is important for soluble expression of BSSα. BSSβ also contains a [4Fe–4S] cluster and is thought to play a role in conformational regulation (Fig. 1*E*) (24, 26, 27). When BSSβ is not associated with the BSS complex, BSSα can adopt an open conformation that allows BSS-AE to install glycyl radical. Once the radical is installed, it has been shown that BSSβ must be added for fumarate addition to occur, which is consistent with its role in securing the GRD in the fully closed conformation (24, 26, 27). In addition to this role, BSSβ plugs the substrate channel, ensuring tight toluene binding in the active site during catalysis (26, 27). BSSβ is currently the only GRE accessory subunit directly implicated in regulation of catalysis.

Like BSS, MASS is also thought to exist as a multi-subunit GRE; however, there are currently no structural data available for any alkyl-SS. The *mas* gene cluster from strain HxN1 was identified in 2008 and found to contain 7 genes (*masA*–*masG*) (5). *masD*, *masC,* and *masG* share sequence homology with *tutD* (gene encoding BSSα), *tutF* (gene encoding BSSγ), and *tutE* (gene encoding BSS-AE), respectively (Fig. 1 *A* and *B*). *masB* and *masE* are thought to encode accessory subunits that could be involved in alkane activation. *masA* encodes a putative acyl CoA dehydrogenase and *masF* encodes a putative transposase (5). Since the discovery of this gene cluster, Cirino and coworkers have demonstrated the importance of the *masB* and *masE* genes in formation of alkylsuccinates through heterologous expression in *E. coli* (30). Here, we present a structure of the MASS complex solved by single particle cryogenic electron microscopy (cryo-EM). Based on structural similarity to BSS, we have named the protein products of *masD*, *masC*, and *masB*, MASSα, MASSγ, and MASSβ, respectively. The protein product of *masE* resulted in a subunit with no known sequence or structural identity to any currently characterized GRE, and thus we have named this subunit MASSδ. The structure of the MASS complex bound to fumarate has allowed us to propose roles for the accessory subunits and putative substrate gating mechanisms within MASS.

## Results

### A Complex Containing MASSαβγδ can be Heterologously Expressed and Purified.

Although MASS from strain HxN1 was originally predicted to consist of three distinct subunits like BSS, (5) it is now thought to include four subunits (*masBCDE*) (30). The large, catalytic subunit, MASSα (94.7 kDa, *masD*) shares 33% sequence identity (31) with BSSα. Similarly, MASSγ (7.0 kDa, *masC*) resembles BSSγ, sharing 31% sequence identity and containing a CX_2_CX_n_CX_n_C motif that coordinates a [4Fe–4S] cluster in BSSγ (29). BSSαγ has been shown to form a stable heterotetramer, (26, 27, 29) and thus we sought to initially characterize the homologous MASSαγ complex. A 6xHis-tag was added to *masD* and was coexpressed with *masC* in *E. coli*. Anaerobic purification by immobilized metal affinity chromatography (IMAC) of MASSαγ resulted in soluble brown protein, which indicated that MASSγ likely binds a [4Fe–4S] cluster like BSSγ. MASSαγ displayed different oligomeric states than the BSSαγ complex when purified by size exclusion chromatography (SEC). MASSαγ purifies as a mixture of species, but primarily as a heterodimer [MASS(αγ)] (*SI Appendix*, Fig. S1*A*). Multiple expression and purification conditions were screened in an attempt to stabilize the homodimer of heterodimers [MASS(αγ)_2_], but only stabilization of the monomer of heterodimers was observed.

We next tried coexpression of *masC* and *masD* with *masB* and *masE*, neither of which share sequence similarity to any component of the BSS complex. When all four subunits are coexpressed, the ratio of MASSα dimer to MASSα monomer increases by SEC; however, much smaller yields of protein are obtained (<1 mg protein per L culture, *SI Appendix*, Fig. S1*A*). By SDS-PAGE gel, it appears that all three smaller subunits bind to the form of the complex that contains dimeric MASSα, but the protein product of *masB* cannot be observed in the monomeric MASSα fractions (*SI Appendix*, Fig. S1*A*). The complex that contains dimeric MASSα dissociates over a short period of time (*SI Appendix*, Fig. S1*B*). As our goal was to structurally characterize the entire complex to determine subunit architecture, we reasoned that single particle cryoelectron microscopy (cryo-EM) would be the most practical technique for this low-yielding, unstable complex.

### EM Maps of the MASS Complex can be Obtained Through Anaerobic Sample Preparation.

We assessed the feasibility of using EM to structurally characterize MASS through negative staining. Negative stain grids of the SEC fractions corresponding to dimeric MASSα containing unknown ratios of the β, γ_,_ and δ subunits were made both aerobically and anaerobically to test the oxygen sensitivity of the complex, as we suspected it contained multiple Fe cofactors. Small data sets for each condition (aerobic vs. anaerobic) were collected and images were processed through 2D classification. By comparison of 2D classes, the anaerobic samples contained more side views of the MASS dimeric complex and displayed less heterogeneity (*SI Appendix*, Figs. S2 and S3). More images were collected on the anaerobically prepared grids and a 3D reconstruction was obtained (*SI Appendix*, Fig. S4). This reconstruction is the approximate size of a dimeric MASS complex as judged by the fit of the map to the structure of BSS(αβγ)_2_ (PDB ID: 5BWE) (27) (*SI Appendix*, Fig. S5*A*).

Based on the negative stain results, we prepared grids for cryo-EM in an anaerobic chamber following the same protein purification protocol. To assess the quality of the grids, we collected a small dataset, which resulted in an EM map that processed to 7 Å resolution (*SI Appendix*, Table S1 and Fig. S6). When BSS(αβγ)_2_ is modeled into this map, one of the BSSα subunits has a good fit, but the other side of the map is missing density for a large portion of the C-terminus (which includes the glycyl radical domain) as well as the BSSβ subunit (*SI Appendix*, Fig. S5*B*). Both BSSγ subunits fit into density, and there is extra density that corresponds to the termini and a [4Fe–4S] cluster that were not resolved by X-ray crystallography (*SI Appendix*, Fig. S5*C*). The region where BSSβ binds in the BSS complex poorly fits the observed MASS density; in addition, a notable amount of extra density surrounds the modeled BSSβ subunit (*SI Appendix*, Fig. S5*D*). We used these grids to collect a high-resolution dataset that yielded a map to 2.8 Å overall resolution (*SI Appendix*, Figs. S7 and S8 and Table S1). The final map ranged from 2.7 Å resolution in the core of the MASSα subunit to 4.1 Å resolution on the side of the map with regions of MASSα missing (*SI Appendix*, Fig. S9).

### High-Resolution EM Map Affords a Model for an Asymmetric MASSα_2_βγ_2_δ Complex.

**Fig. 2.**
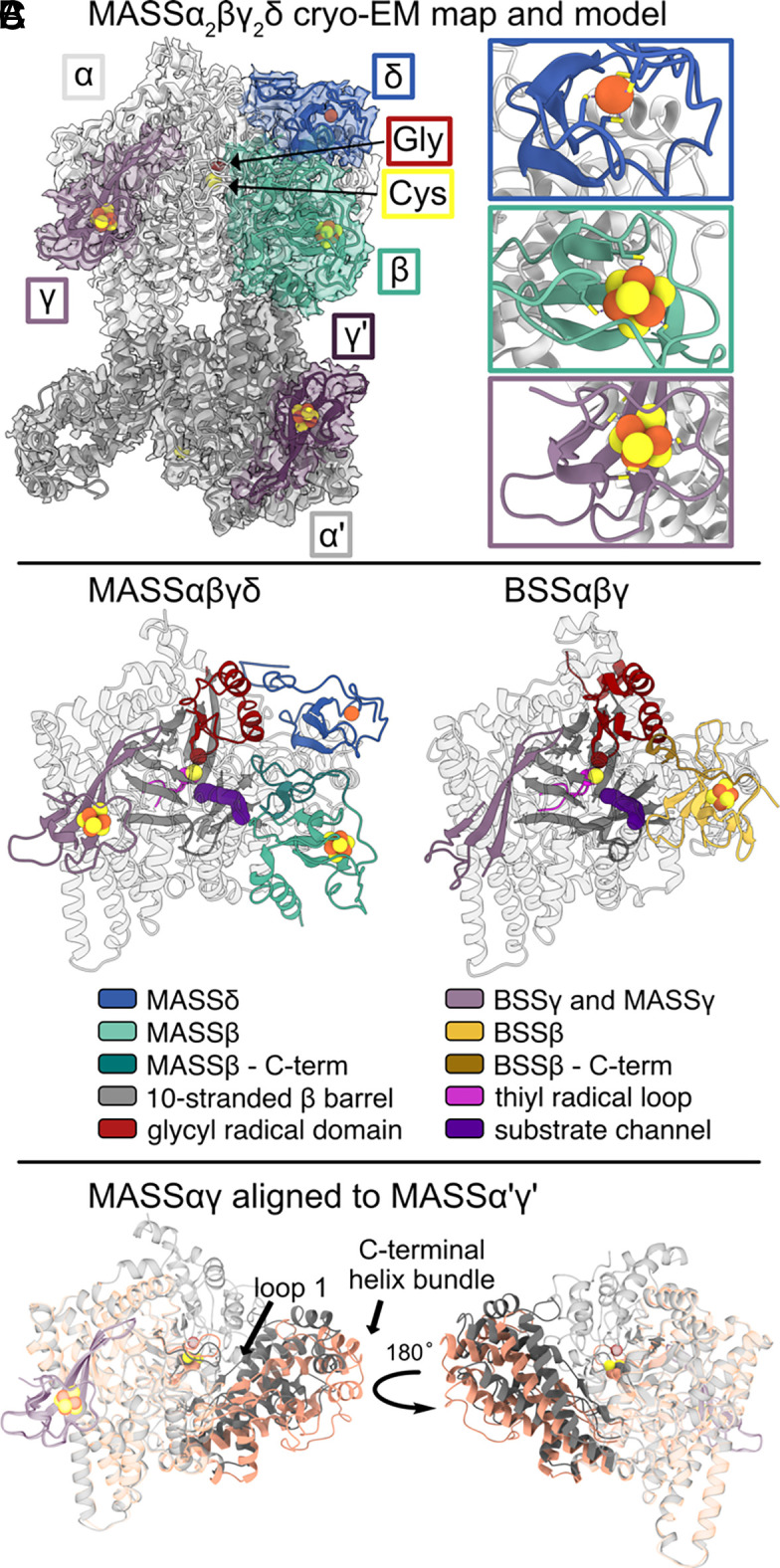
Cryo-EM reveals a molecular model for the MASS complex. (*A*) An EM map to 2.8 Å resolution was used to build a model of an asymmetric MASSα_2_βγ_2_δ complex (*Left*, subunits labeled accordingly). The essential glycine residue is shown as a red sphere and the S atom of the catalytic cysteine residue is shown as a yellow sphere. A single Fe ion is shown as a sphere in the δ subunit (*Right*, blue box). 4Fe–4S clusters are shown as spheres in the β (*Right*, green box), γ (*Right*, purple box), and γ′ subunits. (*B*) Comparison of MASSαβγδ (*Left*) and BSSαβγ (*Right*, PDB ID: 5BWE). (*C*) MASSαγ (gray) and MASSα′γ′ (salmon) overlaid. Large movements are observed in the C-terminal helix bundle and loop 1.

We were able to build a molecular model for a MASSα_2_βγ_2_δ complex using the high-resolution EM map (Fig. 2*A* and *SI Appendix*, Fig. S10). MASSα forms a dimer with an interface similar to other GREs (*SI Appendix*, Fig. S11). One MASSα protomer is more complete than the other (MASSα: residues 15 to 839, MASSα′: residues 36 to 677, missing density for loops 375 to 380 and 588 to 593). Both MASSα and MASSα' bind one MASSγ protomer in a similar manner as BSSα binds BSSγ (Fig. 2*B*). One protomer of MASSβ and one of MASSδ are also bound to MASSα; however, there is no density for either of these subunits on MASSα′. We previously observed that BSSα becomes less well ordered in the absence of BSSβ (26, 27), suggesting that the disordered region of MASSα′ observed here may be due to the lack of MASSβ and MASSδ binding on that side of the MASSα dimer. Although we cannot rule out that the disorder in MASSα′ is due to a cryo-EM artifact such as denaturation at the air–water interface, we believe that both the MASSαβδγ and MASSα′γ sides of this structure represent functionally relevant states, with comparison revealing the stability afforded to MASSα chain by the presence of MASSβ and MASSδ. On the MASSαβδγ side, MASSβ binds to a similar location on the surface of MASSα as BSSβ does on BSSα; however, MASSβ extends much farther down the MASSα subunit toward the dimer interface (Fig. 2*B*). MASSδ binds close to the glycyl radical domain (GRD) and close to MASSβ (Fig. 2*B*).

MASSα displays the common GRE fold: a 10-stranded β/α-barrel that is composed of two antiparallel 5-stranded β-sheets that are surrounded by helices (Fig. 2*B* and *SI Appendix*, Fig. S12). The Cys and Gly loops are located in the center of this barrel. The Cys loop follows the final β-strand of the first half barrel (strand 5) and the Gly loop follows the final β-strand of the second half barrel (strand 10) (*SI Appendix*, Fig. S12). In contrast to MASSα, a large portion of the C-terminus of MASSα′ is disordered (either through conformational flexibility or through denaturation during sample preparation). The region of MASSα′ that could not be modeled (residues 678 to 839) contains the glycyl radical domain, which was shown to be more conformationally flexible than the barrel core in BSS (26). Clear density is observed for the first 5-stranded β/α-half barrel of MASSα′ (*SI Appendix*, Fig. S13*A*). For the second 5-stranded β/α-half barrel of MASSα′, only 2 of the 5 β-strands could be modeled (β6 and β7, *SI Appendix*, Fig. S13*B*). These strands connect to a C-terminal helix bundle that was observed to move in BSS when BSSβ is absent (*SI Appendix*, Fig. S12). The resolution is lower within residues 602 to 677 of MASSα′; however, a C-terminal helix bundle similar to that in BSS could be placed within this density. The aligned structures of MASSα and MASSα′ indicate that the C-terminal helix bundle of MASSα′ has moved outward in a clamshell like motion (Fig. 2*C*), similar to the movement observed when comparing the structures of BSSαγ to BSSαβγ. The movement of this bundle has been previously proposed to accompany the glycyl radical loop movement out of the active site, making it accessible to BSS-AE.

**Fig. 3.**
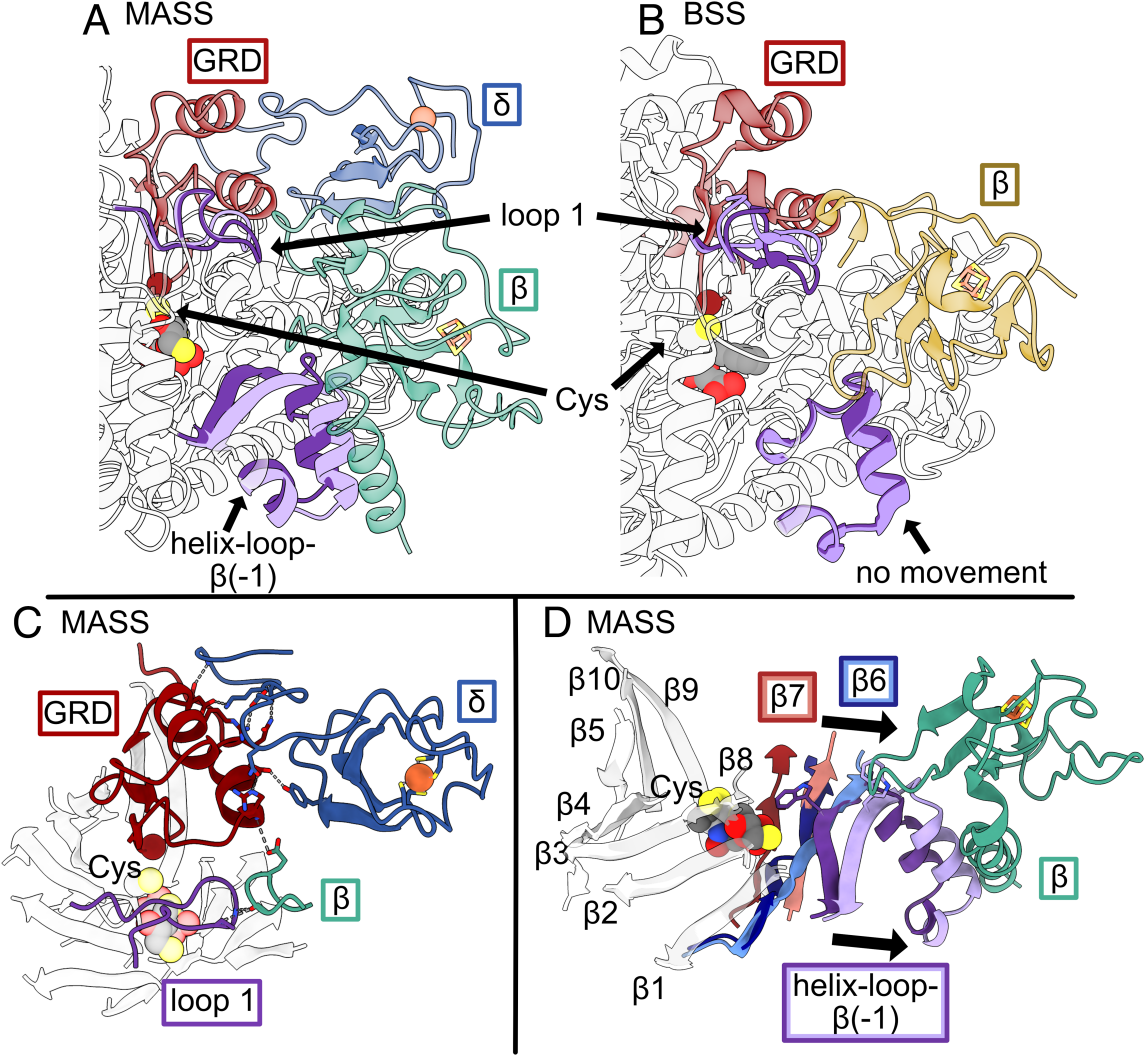
Accessory subunit binding to MASSα. (*A*) MASSα (with MASSβ and MASSδ bound) and MASSα′ are aligned. Loop 1 and helix-loop-β(−1) motifs are highlighted in purple (MASSα shown as dark purple and MASSα′ shown as light purple). MASSβ is shown in green, MASSδ is shown in blue, and the glycyl radical domain (GRD) is shown in red. When MASSβ is not bound to MASSα, movement is observed in loop 1 and helix-loop- β(−1). (*B*) Structures of BSSα with (PDB ID: 5BWE) and without (PDB ID: 5BWD) BSSβ are aligned. Loop 1 and the region corresponding to the helix-loop-β(−1) motif in MASS are highlighted in purple (BSSαβγ shown as dark purple and BSSαγ shown as light purple). Movement is observed in loop 1 when the two structures are compared; however, no movement is observed in the region corresponding to the MASS helix-loop-β(−1) motif. The catalytic Cys residue is labeled “Cys” in panels *A* and *B*. (*C*) The interactions between loop 1 of MASSα, the GRD of MASSα, MASSβ, and MASSδ are shown as sticks. The C-terminus of MASSδ engages in many electrostatic interactions with the GRD. (*D*) The β-barrel for MASSα and partial β-barrel for MASSα′ is shown, with each strand labeled accordingly. MASSα corresponds to darker colors and MASSα′ corresponds to lighter colors. Strands 1 to 5 align well between the protomers. Strands 8 to 10 could not be modeled for MASSα′. Strands 6 (blue) and 7 (red) shift over by one strand, along with β(−1), when MASSβ is not bound, resulting in opening of the active site as indicated by arrows. W185, shown as sticks, is positioned toward the active site in MASSα (dark purple) and away from the active site in MASSα′ (light purple).

In addition to the C-terminal helix bundle, other more localized changes are observed between the α and α′ subunits. Movement is observed in residues 48 to 57, which correspond to “loop 1” (residues 55 to 65) in BSSα (Fig. 3*A*). In BSS, loop 1 adopts two conformations (Fig. 3*B*), which are regulated by BSSβ. The movement between loop 1 in MASS and BSS differs in that BSSβ appears to push loop 1 in toward the active site, whereas MASSβ makes an interaction with loop 1 that pulls the loop toward itself (Fig. 3 *A*–*C*).

Another region that is distinct between MASSα and MASSα′ is a small helix-loop-strand (residues 168 to 189) that immediately precedes β1 (residues 193 to 198) of the first half barrel (Fig. 3 *A* and *D* and *SI Appendix*, Fig. S12). In MASSα, the strand preceding β1 [termed β(−1)] (Fig. 3*D*, dark purple) is not part of the half barrel but does interact with β6 (Fig. 3*D*, dark blue and *SI Appendix*, Fig. S12). Residue Trp185 of the β(−1) strand is positioned such that the side chain is protruding into the active site (Fig. 3*D*). In MASSα′, MASSβ is not bound, which allows the helix of the helix-loop-β(−1) to move away from the barrel (~5 Å, Fig. 3*D*). The strand moves away from the active site with the helix, and Trp185 flips out of the active site. β6 and β7 shift as well to maintain a sheet with the strand. These movements altogether result in a complete 10-stranded barrel that resembles other GRE barrels in MASSα (when β is bound), and a shifted half barrel in MASSα′ (when β is not bound). When BSSαγ is compared to BSSαβγ, no change in this region is observed (Fig. 3*B*).

### MASSγ and MASSβ Adopt HiPIP-Like Folds; MASSδ Adopts a Rubredoxin-Like Fold.

**Fig. 4.**
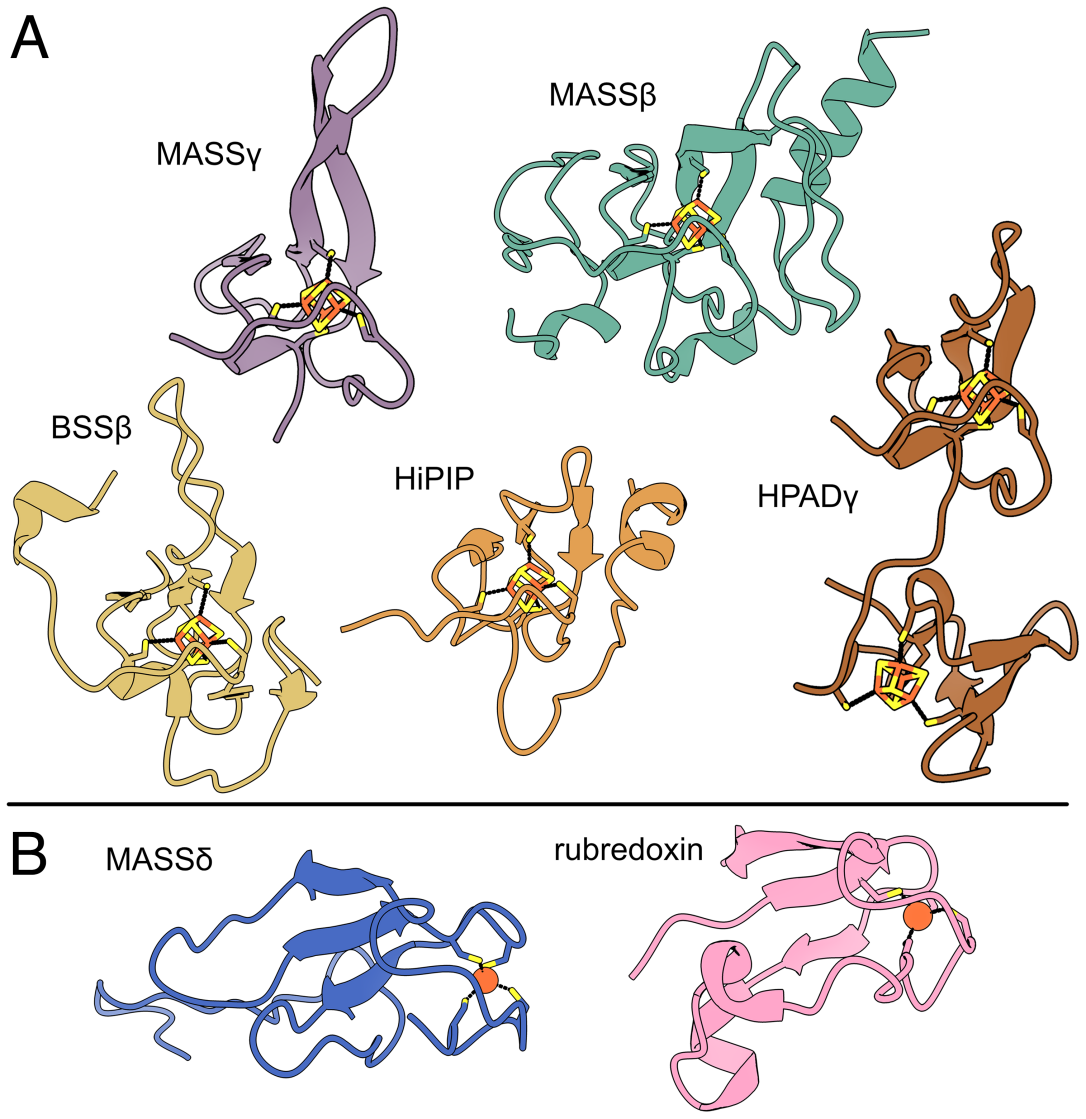
Comparison of GRE accessory subunits. (*A*) 4Fe–4S cluster-binding accessory subunits MASSγ, MASSβ, BSSβ (PDB ID: 5BWE), and HPADγ (PDB ID: 2YAJ) adopt a fold resembling high potential iron-sulfur proteins (HiPIP, PDB ID: 1ISU). 4Fe–4S clusters and coordinating Cys residues are shown as sticks. (*B*) MASSδ coordinates a single Fe ion instead of a 4Fe–4S cluster, making it unique among the characterized GRE accessory subunits. The fold of MASSδ resembles rubredoxins (PDB ID: 6RXN). The Fe ions are shown as orange spheres and the coordinating Cys residues are shown as sticks.

As noted above, MASSγ shares moderate sequence identity to BSSγ (31%). When crystal structures of BSSγ were solved in complex with BSSα, residues 1 to 10 and 48 to 60, along with the [4Fe–4S] cluster, were missing from the crystal structure. When the cryo-EM structure of MASSαβγδ is aligned with BSSαβγ, MASSγ, and BSSγ overlay well, aside from a loop (residues 13 to 19 in MASSγ) that is flipped in a different direction (*SI Appendix*, Fig. S14*A*). Both MASSγ and BSSγ cover a hydrophobic patch on their respective α subunits (*SI Appendix*, Fig. S14 *B* and *C*). Most of MASSγ could be modeled from the cryo-EM map (residues 2 to 54), including the [4Fe–4S] cluster (*SI Appendix*, Fig. S15 *A* and *B*). As expected, MASSγ adopts a fold similar to HPADγ, BSSβ, and high potential iron-sulfur proteins (HiPIP) (Fig. 4*A*).

Like BSSβ, MASSβ also adopts a HiPIP fold and binds a [4Fe–4S] cluster despite sharing no sequence similarity with any subunits of BSS [Fig. 4*A* and *SI Appendix*, Fig. S16 (25, 27, 32)]. The full MASSβ subunit could be built except the starting methionine (residues 2 to 119). Density consistent with a [4Fe–4S] cluster (*SI Appendix*, Fig. S15*C*) is observed. The cluster is ligated by residues Cys32, Cys35, Cys51, and Cys86, which are part of a CX_2_CX_n_CX_n_C sequence motif. MASSβ and BSSβ have both been shown to be necessary for hydroalkylation (24, 30) and both bind in a similar location on the periphery of α. However, MASSβ is rotated about 90˚ relative to BSSβ, which places its [4Fe–4S] in a different location on MASSα but is equally far from the active site [>30 Å in MASS and BSS, (Fig. 2*B* and *SI Appendix*, Fig. S17)]. In addition to placement on the α subunit, key differences in the structure of MASSβ and BSSβ are observed. The N terminus of MASSβ ends in a helix that binds at the MASSα/MASSα′ interface. This helix could play an important role in the helix-loop-β(−1) movement within MASSα, as it sits on top of the helix of the helix-loop-β(−1) motif (Fig. 3 *A* and *D* and *SI Appendix*, Fig. S12). No such interaction or movement is observed in BSSα (Fig. 3*B*). Another key difference can be observed in the loop containing residues 55 to 74 in MASSβ (residues 45 to 57 in BSSβ) (*SI Appendix*, Fig. S17 *C* and *D*). This loop in BSSβ extends up the α subunit toward the C-terminal helix bundle (where MASSδ is located, *SI Appendix*, Fig. S17 *A* and *B*). In MASSβ, this loop is longer, and wraps around the [4Fe–4S], partially shielding it from solvent. Last, the C-terminus is extended in MASSβ by an extra 19 residues. The C-terminus of BSSβ contacts the GRD and loop 1 in the α subunit, the latter of which appears to move in concert with the GRD (Fig. 3*A*). A loop within the C-terminus of MASSβ also contacts the GRD and loop 1 in the α subunit (Fig. 3*C*).

Like MASSβ, MASSδ has no sequence similarity with any subunits of BSS and has been shown to be necessary for activity when MASS is expressed in *E. coli* (30). Residues 1 to 69 (of 71) in MASSδ were modeled into the cryo-EM map, showing the four Cys residues (Cys3, Cys6, Cys32, and Cys35) of a CX_2_CX_n_CX_n_C motif arranged to bind a metal or cluster. However, instead of finding density for the predicted [4Fe–4S] cluster, the density was consistent with the presence of a single Fe ion (Fig. 4*B* and *SI Appendix*, Fig. S15*D*). Furthermore, when we compare the overall fold of MASSδ to that of other known proteins, we observe that MASSδ has a rubredoxin-like fold, a fold associated with the binding of a single Fe ion [Fig. 4*B* (33)]. MASSδ is composed of this rubredoxin-like domain and a ~20 residue C-terminal tail. MASSδ is positioned on MASSα and makes contacts with the neighboring MASSβ subunit through the rubredoxin-like domain (Fig. 3*A*). The C-terminal tail of MASSδ makes extensive interactions with the GRD of MASSα (Fig. 3 *A* and *C*).

### Fumarate and Dithiothreitol are Bound Within the Active Site.

**Fig. 5.**
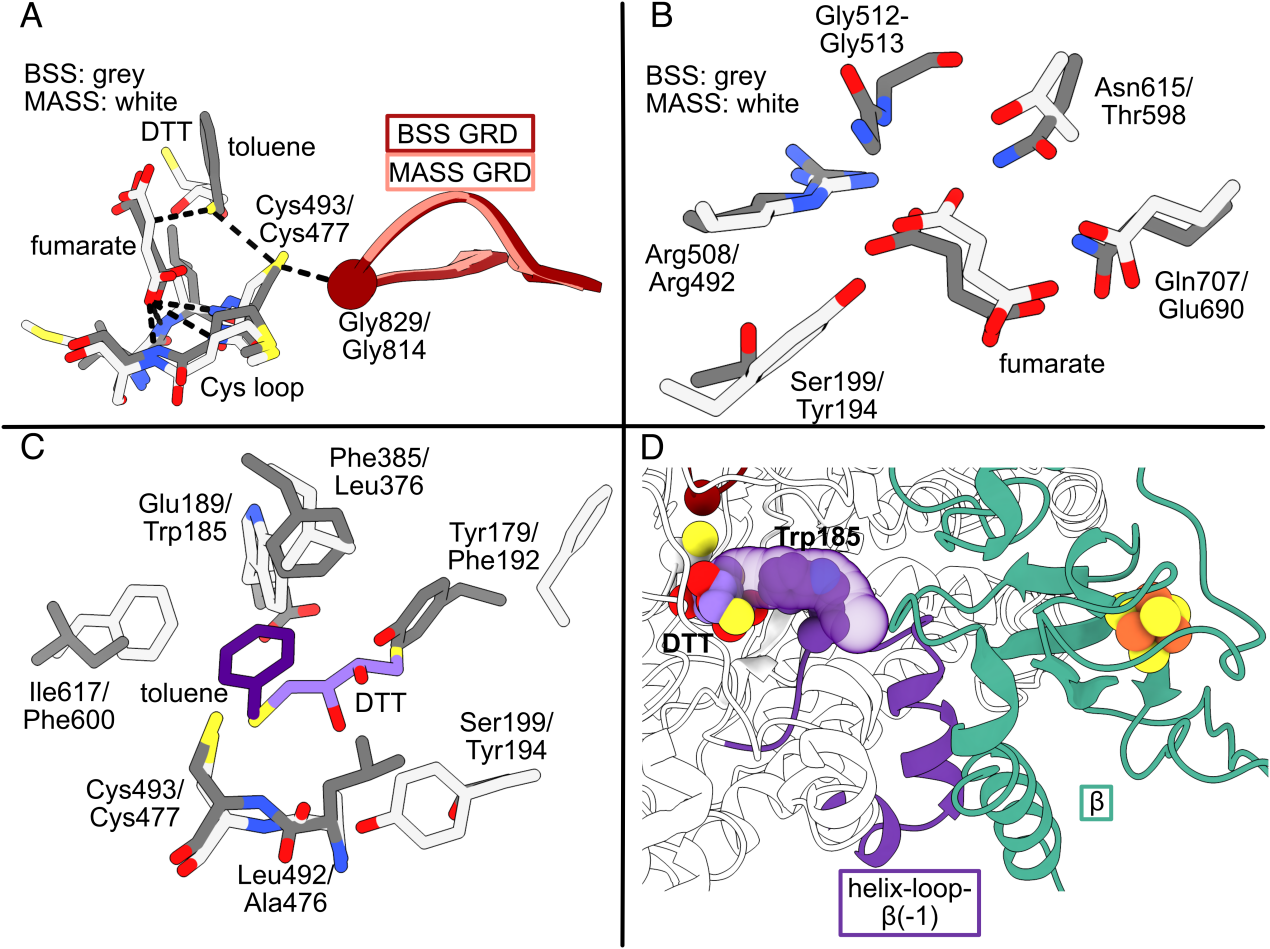
Active site comparison of BSS and MASS. BSSαβγ, shown as darker colors, (PDB ID: 5BWE) was aligned to MASSαβγδ, shown as lighter colors. (*A*) The active sites of BSS and MASS are shown, including the Gly loop, Cys loop, and bound small molecules. The Gly loops of BSSαβγ and MASSαβγδ (dark red and light red, respectively) overlay well. Gly radical can abstract H atom from Cys (Cys493 in BSS and Cys477 in MASS), which can form a hydrocarbyl radical on proximally bound hydrocarbon. The hydrocarbyl radical adds to fumarate to form a succinyl radical. The hydrocarbon is known to be toluene for BSS and *n*-alkanes for MASS. No alkane was present in our freezing conditions; however, density was observed in the hydrocarbon-binding site that could be modeled as DTT. A carboxylate of fumarate can interact with the oxyanion hole created by the Cys loop in both BSS and MASS. (*B*) Fumarate binding differs between BSS and MASS. The Ser-to-Tyr and Asn-to-Thr substitutions from BSS to MASS result in fumarate shifting slightly. Both Gln707 and Glu690 could act as H-bond donors to fumarate, as the pKa of Glu690 is likely driven up by the surrounding hydrophobic pocket. (*C*) Toluene binding (dark purple) in BSS is compared to DTT binding (light purple) in MASS. (*D*) Small molecule access to the active site via the putative hydrocarbon substrate channel is blocked by MASSβ and by Trp185 in MASS. When MASSβ is not bound, Trp185 swings out of the substrate channel, allowing substrate access, as shown in Fig. 3*D*.

The active site of MASS is located within MASSα’s 10-stranded barrel. The Gly and Cys loops resemble the equivalent loops in BSS and other GREs and display a distance of 4.02 Å between Cys-S and Gly-Cα (Fig. 5*A*). In BSSαβγ, the distance is 3.95 Å (PDB ID = 5BWE). Density was observed for fumarate in a similar location as was observed for the fumarate bound to BSS (*SI Appendix*, Fig. S18*A*). Like BSS, one carboxylate group of fumarate is held in place by an oxyanion hole created by the Cys loop (Fig. 5*A*) whereas the other carboxylate group forms an electrostatic interaction with an active site Arg residue (Arg508 in BSS, Arg492 in MASS, Fig. 5*B*). Although these key binding interactions appear similar, all other interactions between residues and fumarate are different between BSS and MASS (Fig. 5*B*). Residue Gln707 can form a H-bond with fumarate in BSS; however, MASS contains a Glu at this position. The positioning of the Glu side chain in close proximity to the carboxylate of fumarate (2.8 Å) suggests that the pK_a_ of Glu may be shifted to favor protonation, enabling hydrogen bonding with fumarate. Additionally, fumarate is moved ~0.8 Å within the active site, presumably due to the presence of the larger amino acids Tyr194 and Thr598 (Ser199 and Asn615 in BSS, respectively). Lastly, the backbone N–H atoms of Gly512 and Gly513, residues found in a loop at the end of strand β6 in BSS, can H-bond with fumarate. These interactions are absent in MASS.

We did not include *n*-alkane in our conditions for grid plunging, hence we were not expecting anything except for fumarate to be bound in the active site. When we had modeled MASSα and fumarate, we noticed additional density in the active site. Dithiothreitol (DTT) was present in our cryoconditions and could be modeled into the density well (*SI Appendix*, Fig. S18*B*). Given that DTT and *n*-hexane are both six-atom-long chains, we suspect that DTT is occupying the hexane binding site, providing a rough idea of how *n*-hexane might bind. We find that an S–H of DTT is positioned closest to the catalytic Cys residue of MASS (3.9 Å, *SI Appendix*, Fig. S18*B*). When BSS and MASS are aligned, this S–H of DTT lines up with the methyl group of toluene, although the methyl group is slightly closer to its corresponding Cys (3.6 Å) (Fig. 5*C*). Considerable differences are observed between the DTT binding site and toluene binding site of BSS. Residues of MASSα (Phe600 and Trp185) occlude toluene binding as observed in BSS, and a residue of BSS (Tyr179) blocks DTT binding as observed in MASS (Fig. 5*C*). The net result of the residue substitutions is that the substrate binding site in MASS is larger and more elongated than in BSS. Whereas toluene occupies a relatively small pocket, surrounded by Ile617, Phe385, and Tyr179, the MASS binding site extends toward Phe192 (Fig. 5*C*), whose side chain is flipped away from the active site in MASSα, creating a cavity that is large enough to accommodate *n*-hexane and even longer hydrocarbon chains such as *n*-octane (Fig. 5*D*).

In other GREs, including BSS, a conserved substrate channel has been proposed (Fig. 2*B*). When BSSβ is present, a loop blocks the entrance to this putative substrate channel, and it is therefore proposed that BSSβ plays a role in toluene gating. A similar loop is observed in MASSβ (residues 40 to 47). Even though MASSβ is rotated about 90˚ relative to BSSβ, the equivalent loop plugs the channel (*SI Appendix*, Fig. S17 *A* and *B*). In BSS, BSSβ is the only barrier to toluene entering the active site; however, in MASS, Trp185 blocks the substrate channel as well (Fig. 5*D*). In the MASSα′ side of the dimer where MASSβ is absent and no substrate is bound, this Trp185 residue flips out of the substrate channel (*SI Appendix*, Fig. S19).

## Discussion

The molecular details of anaerobic alkane activation, a process fundamentally distinct from its aerobic counterpart, remain poorly understood. In the absence of oxygen, GREs catalyze the formation of new C–C bonds between alkanes and fumarate, activating them for further microbial metabolism (1, 2). Structural studies of BSS, an enzyme that activates toluene for degradation, have provided significant insights into aromatic hydrocarbon activation; (26, 27) however, the subunit architecture of alkane-activating GREs was proposed to differ from that of BSS (30). Here, we present the structure of MASS from *Azoarcus* strain HxN1, which activates C6–C8 *n*-alkanes. Although the overall fold of the catalytic subunit is conserved across GREs, the MASS structure reveals notable differences within the active site and adopts distinct conformational states. Additionally, this structure includes an accessory subunit with a rubredoxin-like fold, a motif not observed in any other characterized GREs to date.

Currently known GREs and newly discovered aminoacyl radical enzymes (AAREs) (34) typically function without accessory subunits; however, a few GREs, including HPAD, BSS, and MASS, incorporate accessory subunits. In both BSS and HPAD, the γ-subunit (BSSγ and HPADγ) enhances protein solubility during heterologous expression (29, 35, 36). No additional function has been identified for BSSγ, and although HPADγ has been proposed to play a role in glycyl radical reduction, (36, 37) this putative function has not been experimentally confirmed. Our structure reveals that MASS also includes a γ-subunit that is similar in sequence and structure to BSSγ and likely serves to improve enzyme complex solubility like BSSγ (Fig. 4*A* and *SI Appendix*, Fig. S14). Although HPAD consists of only two subunits (α and γ), BSS contains an additional accessory subunit (BSSβ), and MASS contains two additional subunits (MASSβ and MASSδ) with no sequence homology to known GRE subunits. This compositional difference raises key questions: What roles do MASS’s two other accessory subunits play, and are these roles similar to BSSβ?

**Fig. 6.**
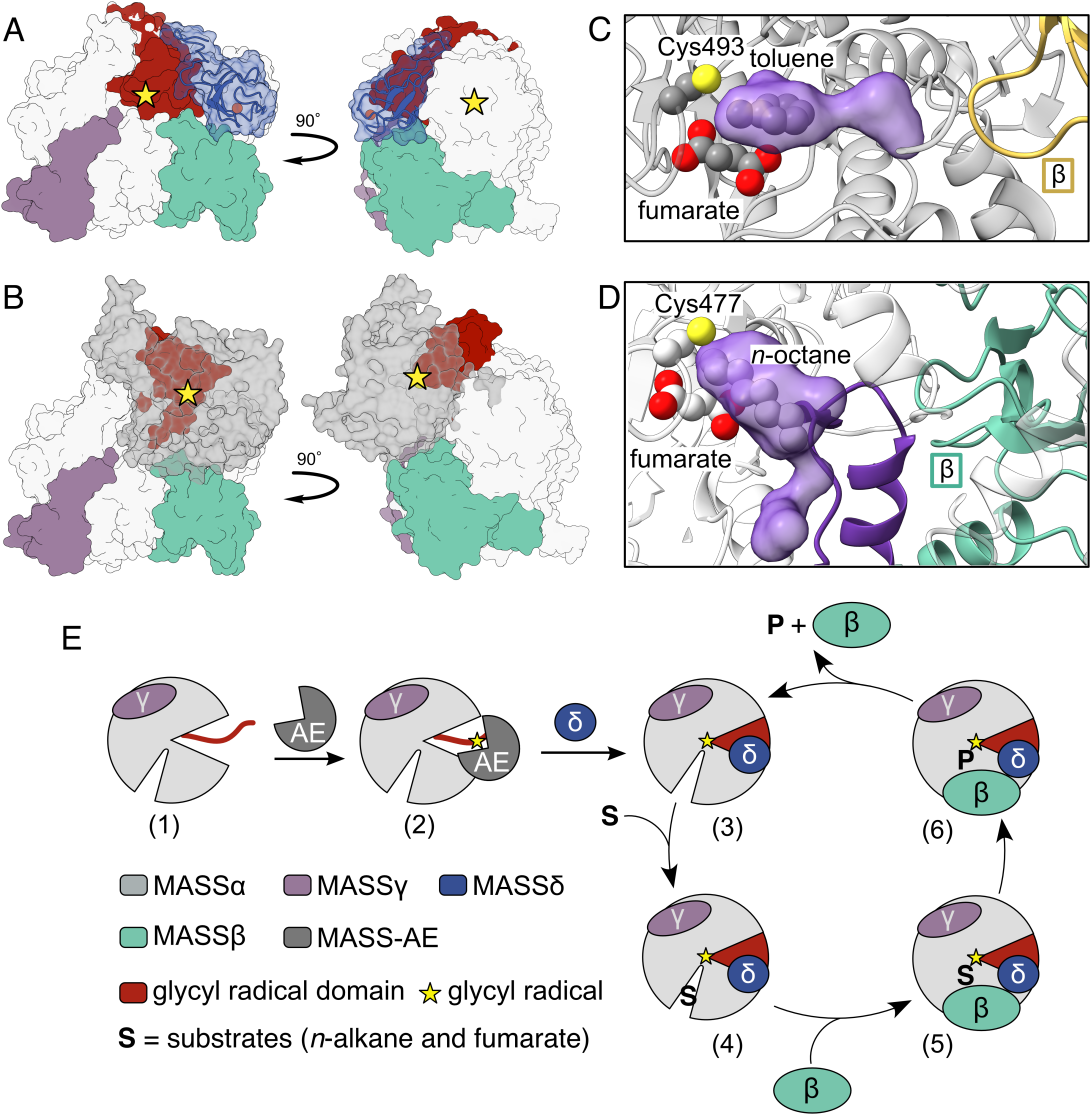
Proposed roles of MASSβ and MASSδ. Based on our structure, we propose that MASSδ and MASSβ each fulfill one of the two roles attributed to BSSβ, where MASSδ could regulate conformational changes of the glycyl radical domain (GRD) upon binding to AE and MASSβ could regulate hydrocarbon entry. (*A*) MASSδ (blue) consists of an N-terminal rubredoxin-like domain and a C-terminal loop that makes extensive contacts with the GRD (red). The Fe-ion-containing rubredoxin-like domain faces outward, away from the interior of MASSα, making a role in electron transfer to the active site unlikely. MASSβ is shown in teal, MASSγ is shown in purple, and star denotes site of the essential glycyl residue. (*B*) When the GRD is modeled into an open conformation to allow AE binding (AE model—AlphaFold, gray), MASSδ must dissociate from MASSα or undergo large movements, as it occupies the same space as the modeled AE. (*C*) BSS active site cavity calculated using HOLLOW from PDB ID: 5BWE with toluene removed. Toluene fits into the calculated pocket, which extends toward a loop in BSSβ—the proposed hydrocarbon substrate channel. (*D*) Potential hydrocarbon binding pockets in MASS were also identified using HOLLOW. *n*-Octane was modeled into the active site based on EM density. The helix–loop–β(−1) region is shown in purple. (*E*) Simplified model for MASS activation and catalysis proposed based on structural data. Key aspects of the mechanism remain unclear, such as whether MASSδ is involved in activation or binds MASSα postactivation, whether MASSβ is bound during activation, and whether MASSβ dissociates or simply rearranges to allow substrate entry.

In BSS, the β-subunit is proposed to serve two roles: gating the requisite rearrangement of glycyl radical domain (GRD) for glycyl radical installation and gating toluene entry (24, 26). We hypothesize that the two roles of BSSβ are divided between these subunits in MASS. Specifically, we propose that MASSβ gates substrate entry, whereas MASSδ regulates GRD conformational changes (Fig. 6).

For glycyl radical installation, the buried Gly loop must rearrange out of the GRE active site to interact with the AE. Previous studies on BSS showed that BSSβ inhibits glycyl radical installation and structural data explained why; BSSβ binds such that GRD movement is restricted and the glycyl loop is secured inside the enzyme core (26, 27). Furthermore, BSSβ protects the GRD from proteolysis, consistent with BSSβ decreasing BSSα flexibility (26). In our cryo-EM structure of MASS, we observe an asymmetric dimer of the α-subunit, with the β- and δ-subunits bound only on one side of the dimer, allowing us to examine how the presence of these subunits affect GRD positioning and overall enzyme flexibility. Comparison of the α-subunits reveals that the GRD is completely disordered when the β- and δ-subunits are absent, consistent with increased GRD mobility in the absence of these subunits. Thus, for both BSS and MASS, one role of subunits appears to be gating GRD motion.

Based on the differences in the GRD between MASSα and MASSα′, it appears that MASSβ and/or MASSδ regulate the conformational changes associated with GRD opening. Structural analysis suggests that MASSδ is the more likely choice, as it makes extensive contacts with the GRD (Fig. 3*C*). The C-terminus of MASSδ must shift to allow the GRD to move out of the active site. Moreover, the entire rubredoxin-like domain of MASSδ must also move, as it occupies the same space that the AE is proposed to bind (Fig. 6 *A* and *B*). It remains unclear whether MASSδ undergoes dramatic conformational changes that allow it to interact with the MASSα–AE complex or whether MASSδ must fully dissociate to permit AE binding.

In addition to differences in GRD density between MASSα and MASSα′, MASS structural analysis reveals distinct conformational variations in regions that remain stationary in BSS structures with and without BSSβ. Notably, we observe movement in the helix-loop-β(−1) motif (Fig. 3 *A* and *B*) and shifting of the second half-barrel of MASSα′ (Fig. 3*D*). We propose that MASSβ, which is notably larger than BSSβ, regulates these movements, and in doing so, regulates alkane entry into the active site. If these movements are indeed physiologically relevant, why are additional conformational changes necessary in MASS as compared to BSS to accommodate its substrate? We suggest two key factors: 1) *n*-alkanes possess far more conformational flexibility than toluene, requiring the enzyme to pay a higher entropic cost to rigidify the substrate for catalysis; 2) though toluene is primarily stabilized by hydrophobic interactions, its π-system can also engage in favorable interactions that further aid binding. As a result, we propose that MASS must open its active site more extensively to bury the alkane within a hydrophobic pocket. This need for a larger entry channel differs from BSS, where toluene is thought to enter through a narrower tunnel before binding in the active site [Fig. 6*C* (38)].

As noted, MASSδ’s position on the GRD suggests it plays a role in the conformational changes associated with glycyl radical installation; however, the use of a rubredoxin-like domain that binds a single Fe ion, rather than a [4Fe–4S] cluster typical of other accessory subunits, remains puzzling. In our hands, MASSδ is more oxygen-sensitive than the other accessory subunits. However, it is unclear if this oxygen-sensitivity is a feature or flaw. A BLAST search on the MASSδ sequence does not show sequence homology to proteins involved in oxygen-sensing or to any characterized proteins, leaving the rationale behind the use of a rubredoxin-like domain enigmatic. Although a rubredoxin fold makes MASSδ distinct from all other characterized GRE subunits, it is noteworthy that alkane hydroxylases (AlkB) also interact with rubredoxin-like proteins, such as AlkG. A recent structure of a fused AlkB–AlkG complex reveals that the rubredoxin-like domain interacts quite differently with AlkB (*SI Appendix*, Fig. S20) than MASSδ does with MASSα (39). The active site of AlkB contains a diiron center that hydroxylates alkanes in the presence of molecular oxygen (39–41). The Fe ion in AlkG is positioned near the diiron center (13.6 Å) and is thought to participate in electron transfer to reduce the AlkB diiron center. In contrast, our structure shows that MASSδ positions its Fe ion away from the active site (39.4 Å), making a role in electron transfer within MASSα unlikely. As noted above, MASSδ and the AE cannot bind simultaneously without MASSδ dissociation or a substantial conformational change (Fig. 6 *A* and *B*). However, it is possible that MASSδ moderates interactions with AE and may even play a role in reducing the AE cluster. If so, it remains an open question why MASS would require this function when no other GRE does.

It has been well established that alkane functionalization via XSSs occurs predominantly at C2 of *n*-alkanes, although minor C1 functionalization of *n*-propane has also been observed (2). The XSS reaction is initiated via H-atom abstraction from the C2 position by the catalytic thiyl radical, a thermodynamically uphill process (BDE of 2˚ C–H bond ~95 to 99 kcal/mol, BDE of S_Cys_–H ~85 to 87 kcal/mol) (42–44). Concerted H-atom abstraction and addition to fumarate is proposed to enable this reaction through formation of a C(sp^3^)–C(sp^3^) bond and a stabilized succinyl radical (Fig. 1*D*) (22). In contrast, H-atom abstraction of toluene by thiyl radical in BSS is more thermoneutral, due to radical stabilization from the benzene ring (BDE ~85 to 90 kcal/mol), (45) and thus could be proceeding through a step-wise mechanism as proposed (9, 10). Although no alkane was included in our grid preparation conditions, we observe density in the expected binding region. This density fits DTT, which was present in our buffer, and has been modeled as such. However, using both this density and prior biochemical knowledge, we can model *n*-octane such that its C2 carbon lies proximal to the catalytic cysteine’s S–H group [Fig. 6*D* (38)]. Inversion at C2 has been observed experimentally, and this is consistent with our structural model when octane is placed in the active site; (22) however, based on this structure alone, we cannot predict the stereochemistry of the products. A high-resolution substrate-bound structure or assays using purified MASS proteins will be required to definitively resolve the stereoselectivity of MASS and its homologs.

Based on our structural data and comparisons with BSS, we propose a model for MASS activation and catalysis (Fig. 6*E*). We hypothesize that the GRD of MASSα is flexible and available for activation in the absence of MASSδ (Fig. 6 *E*, 1). Once the AE has installed the glycyl radical (Fig. 6 *E*, 2), MASSδ binds in the conformation captured in our cryo-EM structure, positioning the radical within the active site (Fig. 6 *E*, 3). We further propose that a conformational change unique to MASS occurs near the substrate channel to expand the entrance and accommodate alkane binding. Upon hydrophobic interactions between the alkane and the substrate channel (Fig. 6 *E*, 4), MASSβ associates to secure the substrate in proximity to the catalytic cysteine residue (Fig. 6 *E*, 5). Following product formation (Fig. 6 *E*, 6), both the product and MASSβ dissociate from MASSα, resetting the enzyme for a new catalytic cycle (returning to Fig. 6 *E*, 3). This model is intentionally simplified, and additional biochemical studies will be necessary to resolve key mechanistic details, including whether MASSβ binds during activation, whether MASSδ directly competes with AE for binding to MASSα or participates in a ternary activation complex, and whether MASSβ dissociates during each catalytic cycle or remains bound and undergoes conformational changes to permit substrate exchange. An additional open question concerns the order of substrate binding. Though both BSS and MASS bind fumarate, their hydrocarbon substrates differ, which is why our discussion has focused primarily on the latter. We hypothesize that fumarate enters the MASSα active site in a manner similar to BSSα, although the timing of this event relative to hydrocarbon binding remains unclear. BSS structures suggest that fumarate binding helps stabilize flexible regions of the active site and can occur independently of BSSβ, unlike hydrocarbon binding (26, 27). By analogy, we propose that fumarate likely binds prior to the hydrocarbon in MASS, though it is not yet known whether it uses the same substrate channel or a distinct entry path.

Overall, our structural analysis of MASS reveals a GRE with three accessory subunits bound to the catalytic subunit simultaneously, more than has been observed for any GRE characterized to date. We speculate that this expanded subunit architecture reflects the dual challenge of binding and activating chemically inert alkanes, which contain no functional handles and possess strong C(sp^3^)–H bonds. In one half of our asymmetric dimer, the absence of two accessory subunits correlates with striking structural differences, leading us to propose distinct roles for these subunits in regulating radical formation, active-site remodeling, and substrate positioning. These findings broaden our understanding of GRE subunit diversity and provide a foundation for future studies into anaerobic hydrocarbon metabolism.

## Materials and Methods

Cloning and plasmid construction, expression, and purification of MASSαβγδ are described in the *SI Appendix*, *Materials and Methods*. Briefly, plasmids containing genes for MASSαγ and MASSβδ were coexpressed in NiCo21(DE3) competent cells (New England BioLabs). MASSα contained an N-terminal His tag, and the complex was purified via immobilized metal affinity chromatography (IMAC), immediately followed by size exclusion chromatography.

Detailed protocols for preparation of negative stain grids aerobically and anaerobically, data collection on resulting grids, and processing of resulting images are described in the *SI Appendix*, *Materials and Methods*.

To prepare cryo-EM grids, a C-flat 1.2 to 1.3 Cu 300 mesh holey-carbon grids (Electron Microscopy Sciences) was glow discharged at −45 mA at 0.39 bar for 1 min. (PELCO easiGlow), cycled into the Coy chamber, and immediately used. Purified MASS (3 μL of 0.8 mg/mL) was added to the grid. After approximately 45 s, the grid was blotted for 4 s with a blot force of −1.0 mm on the blotter that faces the carbon side of the grid and −0.5 mm on the back blotter. After blotting, the grid was plunged into liquid ethane and transferred to a storage button. The same grid was used to collect datasets on a FEI Talos Arctica G2 Cryo 200 kV TEM equipped with a Falcon 3EC camera and a Thermo Fisher Titan Krios 300 kV transmission electron microscope equipped with a Gatan G3i K3 camera. The cryo-EM data collection statistics are summarized in *SI Appendix*, Table S1. Data processing of these two datasets was performed using SPHIRE (46) for frame alignment, CTF estimation, and micrograph selection. Subsequent steps were performed using RELION 3.1 and RELION 4.0 software suites, (47) which were installed through SBGrid (48). The full workflow, FSC curves, and angular distribution plots are described and shown in the *SI Appendix*, *Materials and Methods* and Figs. S6 and S7. The 3DFSC plot (49) and local resolution map of the high-resolution cryo-EM map are shown in *SI Appendix*, Figs. S8 and Fig. S9, respectively.

AlphaFold (50) models of the individual MASSα and MASSγ subunits were docked into the final EM reconstruction using ChimeraX (51). Two molecules of MASSα and two of MASSγ were sequentially docked, resulting in a starting MASSα_2_γ_2_ model. Iterative rounds of model building and refinement of MASSα_2_γ_2_ were done using Coot (52) and Phenix Real Space Refine (53), respectively. The 4Fe–4S cluster and C-terminus of the MASSγ subunits were modeled de novo. Substantial density remained on one side of the dimer. AlphaFold models of MASSβ and MASSδ were iteratively docked into the remaining density using ChimeraX. Regions of MASSβ (residues 93 to 119) and MASSδ (residues 53 to 69) were modeled de novo. The resulting MASSα_2_βγ_2_δ model was refined using Phenix Real Space Refine. Last, extra density in the active site was observed. Fumarate and dithiothreitol were docked into this density. Model quality was evaluated using Molprobity (54) and EMRinger (55). The refinement and model statistics are found in *SI Appendix*, Table S1. The final model contains residues 15 to 839 (of 839) of MASSα (chain A); 36 to 677 (of 839) with loops 375 to 380 and 588 to 593 unmodeled of MASSα′ (chain E); 2 to 54 (of 61) of MASSγ (chain C); 3 to 53 (of 61) of MASSγ′ (chain F); 2 to 119 (of 120) of MASSβ (chain B); and 1 to 69 (of 71) of MASSδ (chain D). Refinement and model statistics are summarized in *SI Appendix*, Table S1. Figures were created with UCSF ChimeraX (51).

## Supplementary Material

Appendix 01 (PDF)

## Data Availability

Coordinates and EM data have been deposited in the protein data bank (PDB) (PDB ID: 9O8U) (56), the electron microscopy data bank (EMDB) (EMD-70238) (57), and the electron microscopy public image archive (EMPIAR) (EMPIAR-12717) (58).
