# Supplementary material for: Structural basis for anaerobic alkane activation by a multisubunit glycyl radical enzyme
Source: Proc Natl Acad Sci U S A. 2025 Aug 4;122(32):e2510389122. doi: 10.1073/pnas.2510389122 (PMC12358834; doi:10.1073/pnas.2510389122)
Supplement: Supplementary file 1 — Appendix 01 (PDF) [file pnas.2510389122.sapp.pdf]

PNAS

Supporting Information for

Structural Basis for Anaerobic Alkane Activation by a Multi-Subunit Glycyl Radical Enzyme

Mary C. Andorfer*[,1,2,3], Talya S. Levitz[2], Jian Liu[1], Ankush Chakraborty[1], Devin T. King-Roberts[4], Delight Nweneka[2], Christa N. Imrich[5], Catherine L. Drennan*[,2,3,5]

[1]Department of Chemistry, Michigan State University, East Lansing, MI 48824
[2]Department of Biology, Massachusetts Institute of Technology, Cambridge, MA 02139
[3]HHMI, Massachusetts Institute of Technology, Cambridge, MA 02139
[4]Department of Biological Engineering, Massachusetts Institute of Technology, Cambridge, MA 02139
[5]Department of Chemistry, Massachusetts Institute of Technology, Cambridge, MA 02139
*To whom correspondence may be addressed Mary C. Andorfer and Catherine L. Drennan
Email: andorfe3@msu.edu (M.C.A.) and cdrennan@mit.edu (C.L.D.)

This PDF file includes:

- Supplementary Material and Methods
- Tables S1 to S2
- Figures S1 to S19
- SI References

Supplementary Materials and Methods

Materials

The genes encoding MASS subunits were purchased from Genewiz as linear gene fragments. Materials and supplies for cloning (including DNA assembly kits, restriction enzymes, and competent cells) were purchased from New England Biolabs. For protein expression and purification, Luria broth, 4-(2-hydroxyethyl)-1-piperazineethanesulfonic acid (HEPES), L-cysteine, iron(II) ammonium sulfate hexahydrate, lysozyme, and sodium fumarate dibasic were purchased from Sigma-Aldrich; benzonase was purchased from EMD Millipore; cOmplete EDTA-free protease inhibitor pellets were purchased from Roche Diagnostics; isopropyl β-d-1-thiogalactopyranoside (IPTG), L-arabinose, kanamycin (Km), ampicillin (Amp), and dithiothreitol (DTT) were purchased from GoldBio; imidazole was purchased from Oakwood Chemicals; TALON resin was purchased from Qiagen. Uranyl acetate used in negative stain grid preparation was purchased from VWR. All grids for negative stain and cryo-EM were purchased from Electron Microscopy Sciences.

Construction of MASS plasmids

A list of constructs created in this study can be found in the Supplemental Information (**Table S2**). MASSαβγδ*:* The genes for the MASSαβγδ complex from *Azoarcus* sp. HxN1 were codon optimized for expression in *E. coli* K12 using the following amino acid sequences: MASSβ (*masB*) – UniProt ID: A9J4K0; MASSγ (*masC*) – UniProt ID: A9J4K2; MASSα (*masD*) - UniProt ID: A9J4K4; and MASSδ (*masE*) - UniProt ID: A9J4K6. All MASS constructs described below were verified through Sanger sequencing by Genewiz and Quintara Biosciences. The genes for MASSγ and MASSα were cloned into a pCOLA-DUET vector. Restriction sites NdeI and XhoI were added to *masC* through PCR amplification using the Q5® Site-Directed Mutagenesis Kit (New England Biolabs) and using primers 5′-ATTATACATATGAGCACATGCAAAGA-3′ and 5′-AATATACTCGAGTCAGTGTGCCTTAG-3′. The resulting *masC* insert was introduced into pCOLA-DUET at multiple cloning site 2 (MCS2) using standard restriction-ligation techniques. Briefly, empty pCOLA-DUET and the amplified *masC* gene were incubated with restriction enzymes NdeI and XhoI (New England Biolabs) and purified over 1% agarose gels using a Qiagen gel extraction kit. The *masC* insert was ligated into MCS2 of pCOLA-DUET and transformed into DH5α cells (New England Biolabs), and isolated plasmid from a single colony was sequenced to verify the pCOLA-DUET- MASSγ construct. The *masD* gene was purchased as two linear fragments containing overlapping regions. Overlaps to the first multiple cloning site (MCS 1) of pCOLA-DUET- MASSγ (which included an N-terminal His-tag) were added through PCR amplification using primers 5′-AGCAGCCATCACCATCATCACCACAGCCAGATGACCGCGACCAGCACCCT-3′ and 5′-CACAAAGGTACCGGTAACGCCCTGCAGACCTTCACGCATGTAACGCGGGC-3′ for the first *masD* fragment and primers 5′-CGTTGCAGCGAGTATGGTAGCTTCAGCCCGCGTTACATGCGTGAAGGTCT-3′ and 5′-CGACCTGCAGGCGCGCCGAGCTCGAATTCGTCAGTGGTGATGATGGTGATGGC-3′ for the second *masD* fragment. The resulting *masD* fragments were gel purified. pCOLA-DUET- MASSγ was linearized with BamH1-HF and gel purified. The two fragments and linearized pCOLA-DUET- MASSγ were assembled using the NEBuilder® HiFi DNA assembly kit. The assembly was transformed into DH5α cells (New England Biolabs) and isolated plasmid from a single colony was sequenced to verify the pCOLA-DUET- MASSαγ construct.

The *masE* and *masB* genes were cloned into a pET-DUET vector. Overlapping regions between *masE* and MCS2 were added by amplification of the purchased *masE* fragment using primers 5′-TACATAATAATACATATGAAATGCACTGAA-3′ and 5′-AGCTGCGCTTCTAGACTAACCCTCAGCAAG-3′ and by amplification of empty pET-DUET vector using primers 5′-CTTGCTGAGGGTTAGTCTAGAAGCGCAGCT-3′ and 5′-TTCAGTGCATTTCATATGTATTATTATGTA-3′. The resulting *masE* fragment and linearized pET-DUET were assembled using the NEBuilder® HiFi DNA assembly kit. The assembly was transformed into DH5α cells (New England Biolabs) and isolated plasmid from a single colony was sequenced to verify the pET-DUET-MASSδ construct. Overlapping regions between *masB* and MCS1 were added by amplification of the purchased *masB* fragment using primers 5′-AGAAGGAGATATACCATGAGCCGCCGTGAC-3′ and 5′-TGCGGCCGCAAGCTTTTATGATTTCTTAATTGA-3′ and by amplification of pET-DUET- MASSδ vector using primers 5′-TCAATTAAGAAATCATAAAAGCTTGCGGCCGCA-3′ and 5′-GTCACGGCGGCTCATGGTATATCTCCTTCT-3′. The resulting *masB* fragment and linearized pET-DUET-MASSδ were assembled using the NEBuilder® HiFi DNA assembly kit. The assembly was transformed into

DH5α cells (New England Biolabs) and isolated plasmid from a single colony was sequenced to verify the pET-DUET-MASSβδ construct.

Purification of MASS proteins

MASSαβγδ: The pCOLA-DUET-MASSαγ and pET-DUET-MASSβδ constructs were transformed together into NiCo21(DE3) competent cells (New England BioLabs) and a single colony was used to make a glycerol stock. Starter cultures were inoculated from glycerol stocks and grown overnight in LB containing 50 µg/mL kanamycin and 100 µg/mL ampicillin at 37 °C at 220 rpm. Expression cultures were inoculated with 10 mL of starter culture per 1 L of LB containing 50 µg/mL kanamycin, 100 µg/mL ampicillin, 150 mg iron(II) ammonium sulfate hexahydrate (CAS: 783-85-9), 47 mg L-cysteine (CAS: 52-90-4), and 160 mg sodium fumarate dibasic (CAS: 17013-01-3). Four liters of culture were grown each round of expression and purification, split into 1 L cultures within 2.5 L flasks. Expression cultures were grown at 37 °C at 220 rpm to an OD600 = 0.8, left to cool at 4 °C without shaking for 30 min, and induced with 1 mM IPTG (GoldBio). Induced cultures were expressed for 5.5 hours at 22 °C at 100 rpm. Cells were pelleted by centrifugation (3,500 rpm for 15 min), flash frozen in liquid nitrogen, and stored at -80 °C until lysis. Cells were cycled into an MBraun while frozen. Cell lysis and purification were performed in an MBraun chamber. All buffers were sparged with argon before use. For lysis of cells, cell paste from 2 L of culture was resuspended in 25 mL lysis buffer (lysis buffer: 50 mM HEPES pH 8.0, 300 mM NaCl, 1 mM fumarate), with an EDTA-free protease inhibitor pellet (cOmplete, Roche Diagnostics), lysozyme (1 mg lysozyme/ml buffer, Sigma Aldrich), and 2 µL benzonase (EMD Millipore). Cells were resuspended by mashing cell paste with a spatula. Resuspended cells were incubated for 30 min at 4 °C, after which cells were sonicated for 2 x 1 min cycles of 2 s on and 15 s off at an amplitude of 10 (Qsonica). Lysate was clarified by centrifugation for 45 min at 28,000 g and subsequently filtered (0.22 µm) before purification by immobilized metal affinity chromatography (IMAC, TALON resin). The MASSαβγδ was purified on TALON resin, which was gravity-packed into a 10 mL plastic spin column (Thermo Scientific™ Pierce™ Centrifuge Columns). The column was equilibrated with buffer (50 mM HEPES pH 8.0, 300 mM NaCl, 1 mM fumarate) before passing clarified cell lysate through by gravity. Columns were washed with buffer (50 mM HEPES pH 8.0, 300 mM NaCl, 5 mM imidazole, 1 mM fumarate) and eluted into new 50 mL falcon tubes with ~2 mL of elution buffer (50 mM HEPES pH 8.0, 300 mM NaCl, 100 mM imidazole, 1 mM fumarate). Only the MASSα subunit contains a His-tag, ensuring accessory subunits in elution fractions were bound to MASSα. The 2 mL of elution buffer was immediately injected onto an anaerobic S200 16/60 column and purified by size exclusion chromatography (50 mM HEPES pH 8.0, 300 mM NaCl, 1 mM fumarate, 1 mM DTT (dithiothreitol)). The peak corresponding to MASS$\alpha_2\beta\gamma_2\delta$ (around 62 mL in **Fig. S1**) was collected anaerobically and immediately used to make negative stain grids and grids for cryo-EM. Purification on TALON resin, size exclusion chromatography, and grid preparation were performed on the same day minimize complex dissociation that occurs over time (**Fig. S1B**). Proteins were used as isolated without 4Fe–4S cluster reconstitutions.

Negative Stain

Grid preparation: Negative stain grids of MASS$\alpha_2\beta\gamma_2\delta$ were prepared anaerobically using freshly purified protein following the purification protocol described above. After collecting the dimer peak from size exclusion, the concentration was determined by measuring the absorbance at 280 nm. MASS$\alpha_2\beta\gamma_2\delta$ was diluted to a final concentration of 10 ng/µL with the following buffer: 50 mM HEPES pH 8.0, 300 mM NaCl, 1 mM fumarate, 1 mM DTT. Carbon-coated 300 mesh copper electron microscopy grids (Electron Microscopy Sciences) were glow discharged outside of the anaerobic chamber for 1 min at -15 mA (PELCO easiGlow). Glow-discharged grids were cycled into the Coy chamber and immediately used. Inside the Coy, 5 µL of MASS$\alpha_2\beta\gamma_2\delta$ was deposited onto the grid. After 45 s, the protein solution was blotted with filter paper (Whatman #1, grade 40) and immediately replaced with a solution of 2% uranyl acetate (VWR). The stain solution was blotted and fresh stain solution was added. This step was repeated and allowed to incubate on the grid for 45 s before final blotting.

Data collection: The negative stain EM data collection statistics are summarized in Table S1. Grids were screened on a FEI Tecnai Spirit transmission electron microscope at the W.M. Keck Microscopy Facility at the Whitehead Institute. Data collections were performed at the Brandeis Electron Microscopy Facility using a FEI Tecnai F20 electron microscope operated at 200 kV on a Gatan US4000 CCD camera using SerialEM software. Images were collected at 62,000 x magnification as 0.8 s exposures with a pixel size of 1.79 Å.

3D reconstruction of MASS$\alpha_2\beta\gamma_2\delta$ *to 25 Å resolution*: An overview of processing workflow for the negative stain data set is shown in **Fig. S4**. For the data set that was used for 3D reconstruction of MASS$\alpha_2\beta\gamma_2\delta$, 77 images were collected, of which 66 were kept for processing in RELION 3.1.[1] From a subset of these micrographs, 996 particles were manually picked and were used to generate a template for automated picking. 41,795 particles were automatically picked, extracted, and 2D classified. Sixty of the 200 2D classes, which corresponded to 30,808 particles, were used to generate initial 3D models *ab initio* (k = 2). The initial model that more closely resembled a typical GRE dimer was used as a reference map in 3D classification of the 30,808 particles (k = 4). Two of the 3D classes (13,432 particles) that more closely resembled dimer were selected and refined to a final map at 24.5 Å resolution.

Comparison of anaerobic vs. aerobic 2D classes: Two data sets were collected to test the oxygen sensitivity of the MASS$\alpha_2\beta\gamma_2\delta$. The anaerobic grid preparation was performed as described above. The aerobic grid preparation was performed as described above, with the exception that they were prepared outside of the Coy chamber under aerobic conditions. Anaerobic and aerobic grids were prepared from the same protein stock in parallel. 54 images were collected of the anaerobic grid and 65 images were collected of the aerobic grid. All 54 images were kept for the anaerobic grid and 60 images were kept for the aerobic grid for processing in RELION 3.1.[1] The images were initially processed together to avoid differences between particle picking between the data sets. Particles were manually picked exhaustively on 3 anaerobic micrographs and 3 aerobic micrographs. Cryolo particle picking software[2] was used to automatically particle pick on the remaining micrographs. Particles from anaerobic micrographs (19,153 particles) and aerobic micrographs (20,355 particles) were separated into different particle stacks and 2D classified.

Cryo-EM plunging

The same grid was used to collect data sets on the Talos Arctica and the Titan Krios. MASS$\alpha_2\beta\gamma_2\delta$ was prepared anaerobically using freshly purified protein following the purification protocol described above. After collecting the dimer peak from size exclusion, the concentration was determined by measuring the absorbance at 280 nm. The protein was concentrated to 0.8 mg/mL and transferred in a sealed vial into a Coy anaerobic chamber, which housed a Cryoplunge3 (cp3, Gatan inc.). A C-flat 1.2-1.3 Cu 300 mesh holey-carbon grid (Electron Microscopy Sciences) was glow discharged at -45 mA at 0.39 bar for 1 min. (PELCO easiGlow), cycled into the Coy chamber, and immediately used. Protein sample (3 μL) was added to the grid. After approximately 45 s, the grid was blotted for 4 s with a blot force of -1.0 mm on the blotter that faces the carbon side of the grid and -0.5 mm on the back blotter. After blotting, the grid was plunged into liquid ethane and transferred to a storage button.

Cryo-EM data collection

The cryo-EM data collection statistics are summarized in **Table S1**. Cryo-EM grids were screened and an initial low-resolution data set was collected at the Cryo-EM Facility at MIT.nano on a FEI Talos Arctica G2 Cryo 200 kV transmission electron microscope equipped with a Falcon 3EC camera at 92,000 x magnification. The collection parameters were as follows: 1.5998 Å/pix, 14 frames, 3.81 electrons/Å^2/frame dose, and underfocus range 1.3 – 3.4 μm. This data set contained 513 movies. These parameters are summarized in **Table S1**.

A high-resolution data set was collected at the Cryo-EM Facility at MIT.nano on a Thermo Fisher Titan Krios 300 kV transmission electron microscope equipped with a Gatan G3i K3 camera at 105,000x magnification. The collection parameters were as follows: 0.832 Å/pix (collected at super-resolution of 0.416 Å/pix), 30 frames, 1.42 electrons/Å^2/frame dose, and underfocus range 1.0 – 2.5 μm. This data set contained 2,462 movies.

Processing of low-resolution cryo-EM data

An overview of processing workflow for the low-resolution cryo-EM data set is shown in **Fig. S6A**. Frame alignment, CTF estimation, and micrograph selection for the low-resolution data set for MASS$\alpha_2\beta\gamma_2\delta$ was carried out using SPHIRE[3]. Individual frames of dose-fractionated exposures were aligned and summed using MotionCorr2, and the defocus of the summed frames was estimated using CTER. Based on drift assessment and CTF assessment performed in SPHIRE, 464 of the 513 micrographs were kept for further processing in RELION 3.1[1]. Particle picking was performed using Topaz.[4] One thousand particles were manually picked from

a subset of micrographs and were used to train the neural-net automated particle picker Topaz. After training, Topaz automatically picked 623,645 particles from the 464 micrographs. These 623,645 particles were narrowed down to 400,379 particles by applying a cutoff threshold of -2. All subsequent processing was performed in RELION 3.1. Frame alignment was rerun using RELION's implementation of the MotionCorr2 program, and CTF estimation was rerun using CTFFIND-4.1 within RELION for the motion-corrected micrographs. Particle coordinates from Topaz were imported into RELION, and 400,379 particles were extracted with a box size of 180 pixels. Extracted particles were submitted to reference-free 2D classification (k = 200, mask diameter of 160 Å) and manual subset selection, which resulted in 389,984 particles. These particles were used to generate a 3D initial model *ab initio* using no imposed symmetry (k = 2, mask diameter of 160 Å). The initial model that more closely resembled a dimer was used as a reference map, and the 389,984 particles were subjected to 3D classification (k=8). Particles from classes 5 and 7 were pooled (totaling 95,139 particles) and subjected to 3D refinement with an initial low-pass filter of 60 Å, which resulted in an 8 Å resolution map. A mask was created from the 3D-refined map (low-pass filter of 15 Å) with an initial binarization threshold of 0.0332 extended by 9 pixels with a soft edge of 9 pixels. Combination of the two half-maps along with local B-factor adjustment was performed and FSC plots were generated in RELION. The final masked resolution at FSC = 0.143 was 7.0 Å (**Fig. S6B-C**).

Processing of high-resolution cryo-EM data

An overview of processing workflow for the high-resolution cryo-EM data set is shown in **Fig. S7A**. Cryo-EM data processing of the high-resolution data set for MASS$\alpha_2\beta\gamma_2\delta$ was carried out using RELION 4.0.[1] Individual frames of dose-fractionated exposures were aligned and summed using RELION's implementation of the MotionCorr2 program, and the defocus of the summed frames was estimated using CTFFIND-4.1 within RELION. Based on CTF assessment performed in RELION, 2,368 of the 2,462 micrographs were selected for further processing. RELION's Laplacian-of-Gaussian autopicker was used to exhaustively pick on a subset of 20 micrographs (min. diameter of 80 Å, max. diameter of 200 Å), which resulted in 17,712 particles. Particles were extracted with a box size of 360 pixels and down-sampled to a box size of 180 pixels (1.664 Å/pix). Extracted particles were submitted to reference-free 2D classification (k = 50, mask diameter of 180 Å) and manual subset selection, which resulted in 5,141 particles from 4 classes. Of the 4 classes, 2 represented top views of MASS$\alpha_2\beta\gamma_2\delta$ and 2 represented side views of MASS$\alpha_2\beta\gamma_2\delta$. These 5,141 particles were used for training in Topaz[4] (wrapper within RELION 4.0), after which, 2,515,942 particles were automatically picked using Topaz. Initially, these 2,515,942 particles were narrowed down to 1,004,943 particles by applying a cutoff threshold of -2 and were extracted with a box size of 360 pixels and down-sampled to a box size of 180 pixels (1.664 Å/pix). Extracted particles were submitted to two rounds of reference-free 2D classification using the VDAM algorithm (k = 200, mask diameter of 180 Å, 200 VDAM mini-batches) and manual subset selection, which resulted in 575,073 particles. These particles were used to generate a 3D initial model *ab initio* using no imposed symmetry (k = 2, mask diameter of 200 Å, 100 VDAM mini-batches). The initial model that more closely resembled a dimer was used as a reference map with an initial low-pass filter of 40 Å, and the 575,073 particles were subjected to 3D classification (k=4, mask diameter of 180 Å, 25 iterations). Particles from the best class (class 4; 361,478 particles) were subjected to 3D refinement with an initial low-pass filter of 40 Å (mask diameter of 180 Å), which resulted in a 3.94 Å resolution map. A mask was created from the 3D-refined map (low-pass filter of 20 Å) with an initial binarization threshold of 0.0138 extended by 5 pixels with a soft edge of 7 pixels. Combination of the two half-maps along with local B-factor adjustment was performed and FSC plots were generated in RELION. The final masked resolution at FSC = 0.143 was 3.4 Å. The 361,478 refined particles were re-extracted with a box size of 360 pixels and down-sampled to a box size of 256 pixels (1.17 Å/pix). These particles were used to generate a 3D initial model *ab initio* using no imposed symmetry (k = 2, mask diameter of 190 Å, 100 VDAM mini-batches). The initial model that more closely resembled a dimer was used as a reference map with an initial low-pass filter of 40 Å, and the 361,478 particles were subjected to 3D classification (k=4, mask diameter of 180 Å, 25 iterations). Particles from classes 3 and 4 (263,137 particles) were subjected to 3D refinement with an initial low-pass filter of 40 Å (mask diameter of 180 Å), which resulted in a 3.99 Å resolution map. A mask was created from the 3D-refined map (low-pass filter of 20 Å) with an initial binarization threshold of 0.00992 extended by 5 pixels with a soft edge of 7 pixels. Combination of the two half-maps along with local B-factor adjustment was performed and FSC plots were generated in RELION. The final masked resolution at FSC = 0.143 was 3.48

Å. CTF refinement (estimate anisotropic magnification, CTF parameter fitting, and estimate beamtilt and trefoil) and Bayesian polishing were performed on this map, and another round of 3D-refinement with masking and post processing was performed, resulting in a 2.97 Å resolution map.

In the first round of processing described above, we were stringent on which particles from 3D classification were carried into subsequent rounds of processing because preferred orientation for top views of the complex were so prevalent. We next wanted to see if using our 2.97 Å resolution map as a reference with more particles than we had previously cut would result in a higher resolution map. We went back to our Topaz particle coordinates and extracted with a new cut-off threshold of -3, box size of 360 pixels (down-sampled to a box size of 180 pixels, 1.664 Å/pix). This extraction resulted in 1,432,605 particles. Extracted particles were split into 4 random subsets, and each subset was submitted to reference-free 2D classification using the VDAM algorithm (k = 200, mask diameter of 180 Å, 200 VDAM mini-batches) and manual subset selection. Particle stacks from manual selections were combined and were submitted to another round of reference-free 2D classification and manual subset selection, which resulted in 753,799 particles. These particles were subjected to 3D refinement using the map from the previous refinement with the same pixel size using a low-pass filter of 20 Å. The refined 753,799 particles were re-extracted with a box size of 360 pixels and down-sampled to a box size of 256 pixels (1.17 Å/pix). The particles were refined using the map from the previous refinement with the same pixel size using a low-pass filter of 20 Å. A mask was created from the 3D-refined map (low-pass filter of 15 Å) with an initial binarization threshold of 0.0087 extended by 5 pixels with a soft edge of 7 pixels, and particles were subjected to 3D refinement with this mask. CTF refinement (estimate anisotropic magnification, CTF parameter fitting, and estimate beamtilt and trefoil) was performed on this map. After CTF refinement, a focused mask for the more complete side of the dimer was created (low-pass filter of 15 Å, initial binarization threshold of 0.0075 extended by 5 pixels with a soft edge of 7 pixels) and was used in a 3D classification without alignment of refined particles (k=12, mask diameter of 160 Å, 25 iterations). Particles from 2 of 12 classes were selected (456,512), and the particles from the remaining 10 classes were joined and subjected to 2D classification. Because side views were underrepresented, the side view 2D classes were manually selected (31,277) and combined with the 456,512 particles for a total of 487,789 particles. These particles were subjected to 3D refinement and Bayesian polishing. Another round of 3D classification without alignment was performed (k=12, mask diameter of 160 Å, 25 iterations) with the focused mask (mask focused on MASSα side of the dimer). Particles from the best class (356,678 particles) were subjected to 3D refinement with an initial low-pass filter of 20 Å, which resulted in a 2.8 Å resolution map. Notably, addition of the second two best classes yielded a lower resolution map, so we used only the one best class (356,678 particles) for the final map (**Fig. S7A)**. A mask was created from the 3D-refined map (low-pass filter of 15 Å) extended by 9 pixels with a soft edge of 9 pixels. Combination of the two half-maps along with local B-factor adjustment was performed and FSC plots were generated in RELION. The final masked resolution at FSC = 0.143 was 2.8 Å **Fig. S7B-C**).

Model building of MASSα$_2$βγ$_2$δ

AlphaFold[5] models of the individual MASSα and MASSγ subunits were docked into the EM reconstruction using ChimeraX[6]. Two molecules of MASSα and two of MASSγ were sequentially docked, resulting in a starting MASSα$_2$γ$_2$ model. Iterative rounds of model building and refinement of MASSα$_2$γ$_2$ were done using Coot[7] and Phenix Real Space Refine[8], respectively. For real space refinement, resolution was set to 2.8 Å. The 4Fe–4S cluster and C-terminus of the MASSγ subunits were modeled de novo. Substantial density remained on one side of the dimer. The AlphaFold model of MASSβ was docked into the remaining density using ChimeraX. The C-terminus of MASSβ (residues 93–119) was modeled de novo, as the Alphafold model did not fit the density in this region. The resulting MASSα$_2$βγ$_2$ was refined using Phenix Real Space Refine. Density remained on one side of the dimer. The AlphaFold model of the rubredoxin-like domain of MASSδ (N-terminus) was docked into the remaining density using ChimeraX. The C-terminus of MASSδ (residues 53–69) was built de novo. Iterative rounds of model building and refinement of MASSα$_2$βγ$_2$δ were done. Lastly, extra density in the active site was observed. Fumarate and dithiothreitol were docked into this density. Model quality was evaluated using Molprobity[9] and EMRinger[10]. The final model contains residues 15-839 (of 839) of α (chain A); 36-677 (of 839) with loops 375–380 and 588–593 unmodeled of α' (chain E); 2–54 (of 61) of γ (chain C); 3–53 (of 61) of γ' (chain F); 2-119 (of 120) of β (chain B); and 1-69 (of 71) of δ (chain D). Refinement and model statistics are summarized in Table S1. Figures were created with UCSF ChimeraX[6].

Generation of MASS$\alpha_2\beta\gamma_2$:AE model

The MASS$\alpha_2\beta\gamma_2$:AE model shown in Figure 6 was generated to illustrate a hypothetical binding mode of AE to a putative open conformation of MASS. As no structure of the open state is currently available for any GRE, the putative open conformation of MASS was modelled as previously described for pyruvate formate-lyase.[11] Currently, the only AE structure that has been reported is that of PFL-AE, which was solved in complex with a 7-mer peptide mimic of the glycyl radical loop of PFL (PDB ID: 3CB8).[12] This 7-mer peptide was aligned to the glycyl radical loop of MASS in the putative open conformation. The AlphaFold model of MASS-AE was subsequently aligned with PFL-AE to generate the MASS$\alpha_2\beta\gamma_2$:AE model.

Table S1: Data collection and reconstruction parameters for negative stain and cryo-EM data sets (top). Refinement statistics for MASS$\alpha_2\beta\gamma_2\delta$ high-resolution cryo-EM structure (bottom).

**Data collection**			
Dataset	Negative stain	cryo-EM (low-resolution)	cryo-EM (high-resolution)
Microscope	FEI Tecnai F20	FEI Talos Artica G2	FEI Titan Krios
Camera	Gatan US4000 CCD in integrating mode	Falcon 3EC in counting mode	G3i K3 in super-resolution mode
Acceleration voltage (kV)	200	200	300
Spherical aberration (mm)	1.2	2.7	2.7
Magnification (x)	62,000	92,000	105,000
Pixel size (Å)	1.79	1.5998	0.832 (0.416 super-res)
Defocus range (µm)	1.5 - 0.5	1.3 - 3.4	1.0 - 2.5
Number of frames	-	14	30
Exposure time (s)	0.8	7	6
Total exposure ($e^-/Å^2$)	30	53.34	42.73
Total micrographs collected	77	513	2462
Particles			
Micrographs used for selection	66	464	2368
Total particles	41,795	400,379	1,432,605
Automation software	SerialEM[13]	EPU	EPU
**Reconstruction**			
Software	RELION-3.1[1]	SPHIRE,[3] Topaz,[4] RELION-3.1[1]	RELION-4.0[1]
Final number of particles	13,432	95,139	356,678
Symmetry imposed	none	none	none
Unmasked resolution at 0.143 FSC (Å)	24.5 Å	8.0	2.8
Masked resolution at 0.143 FSC (Å)	-	7.0	2.8
Local resolution range (Å)	-	-	2.67–4.15

**Model Refinement of MASS$\alpha_2\beta\gamma_2\delta$ high-resolution cryo-EM structure**	
Refinement software	Phenix[14]
Refined model resolution at 0.143 FSC (Å)	2.8
$CC_{volume}/CC_{mask}/CC_{box}$	0.73 / 0.74 / 0.66
Mean CC for ligands	0.77
Model composition	
Non-hydrogen atoms	13,972
Residues	1,746
4Fe–4S clusters (SF4)	3
1Fe–0S cluster (FE)	1
Fumarate (FUM)	1
Dithiothreitol (DTT)	1

Rms deviations<br>Bond lengths (Å)<br>Bond angles (°)	 0.005<br>0.977
MolProbity Score	1.72
Clash score	10
Rotamers outliers	8 (0.5%)
Ramachandran<br>Favored<br>Allowed<br>Outliers	 1679 (97%)<br>51 (2.9%)<br>1 (0.1%)
CaBLAM outliers (%)	1.81
EMRinger score	3.31
Average Q-score	0.5140
B-factors (min/max/mean)	
Protein	30 / 226.6 / 72.76
4Fe–4S clusters (SF4)	58.23 / 87.02 / 77.42
1Fe–0S cluster (FE)	99.07 / 99.07 / 99.07
Fumarate (FUM)	59.54 / 59.54 / 59.54
Dithiothreitol (DTT)	68.22 / 68.22 / 68.22

Table S2: List of constructs created in this study.

Construct name	Parent vector	Gene(s)	Tag(s)
pCOLA-DUET-**MASSγ**	pCOLA-DUET	*masC* (MCS2)	None
pCOLA-DUET-**MASSαγ**	pCOLA-DUET	*masD* (MCS1)<br>*masC* (MCS2)	N-terminal His-tag on MASSα (MGSSHHHHHHSQ)
pET-DUET-**MASSδ**	pET-DUET vector	*masE* (MCS2)	None
pET-DUET-**MASSβδ**	pET-DUET vector	*masB* (MCS1)<br>*masE* (MCS2)	None

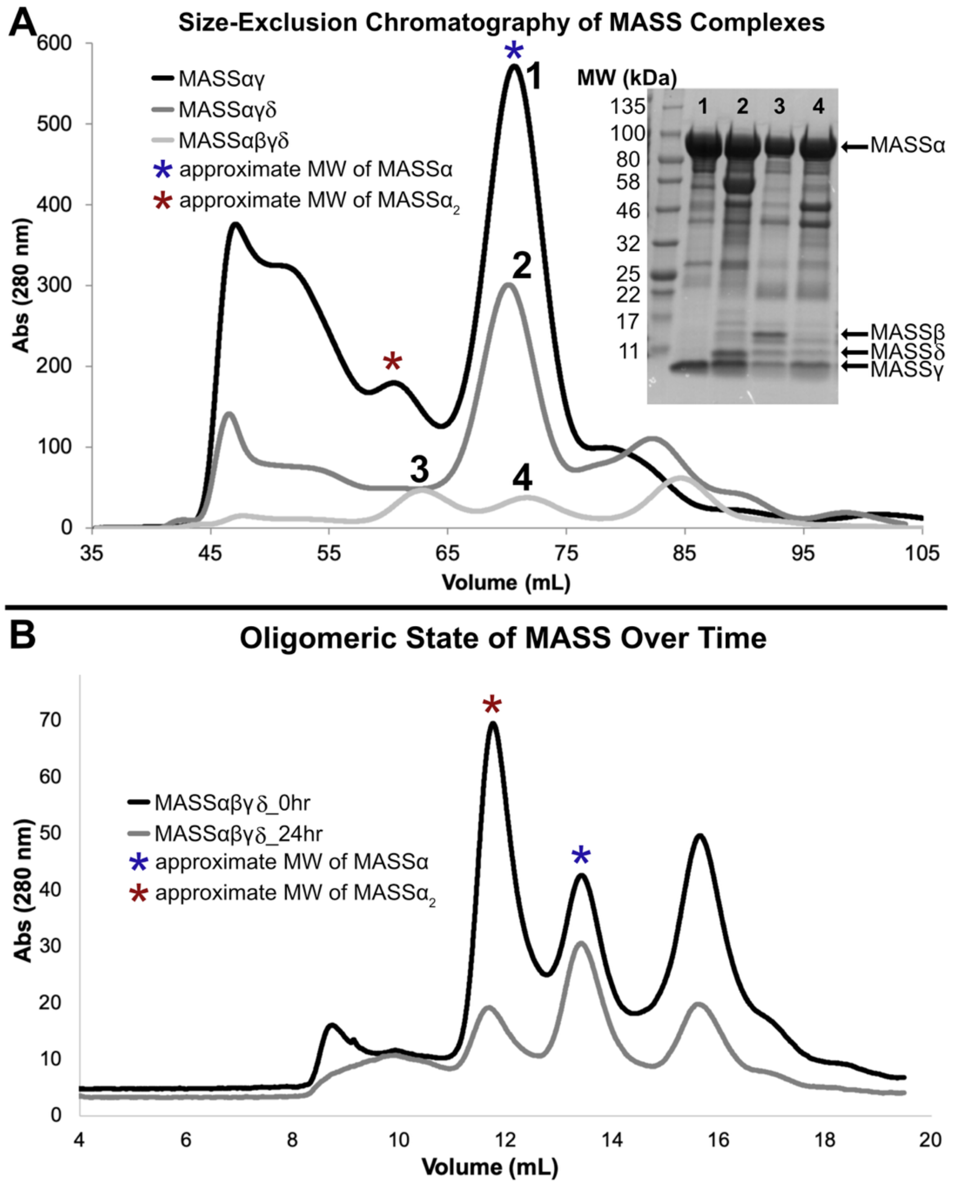

Figure S1: Characterization of MASS complexes by SEC. A) The genes for MASSαγ, MASSαγδ, and MASSαβγδ were over-expressed and TALON purified (only the MASSα subunit is His-tagged). Following purification, the elution fraction was submitted to SEC. The 3 traces are shown above. In all cases, a mixture of oligomers is observed. Entries in gel: **1** = MASSαγ (peak around 70 mL), **2** = MASSαγδ (peak around 70 mL), **3** = MASSαβγδ (peak around 60 mL), **4** = MASSαβγδ (peak around 70 mL). The peaks corresponding to the gel entries are labeled 1–4. B) Oligomeric state of the MASSαβγδ complex over time. The ratio of MASSα dimer to MASSα monomer reduces significantly over a period of 24 hours at 4 ˚C.

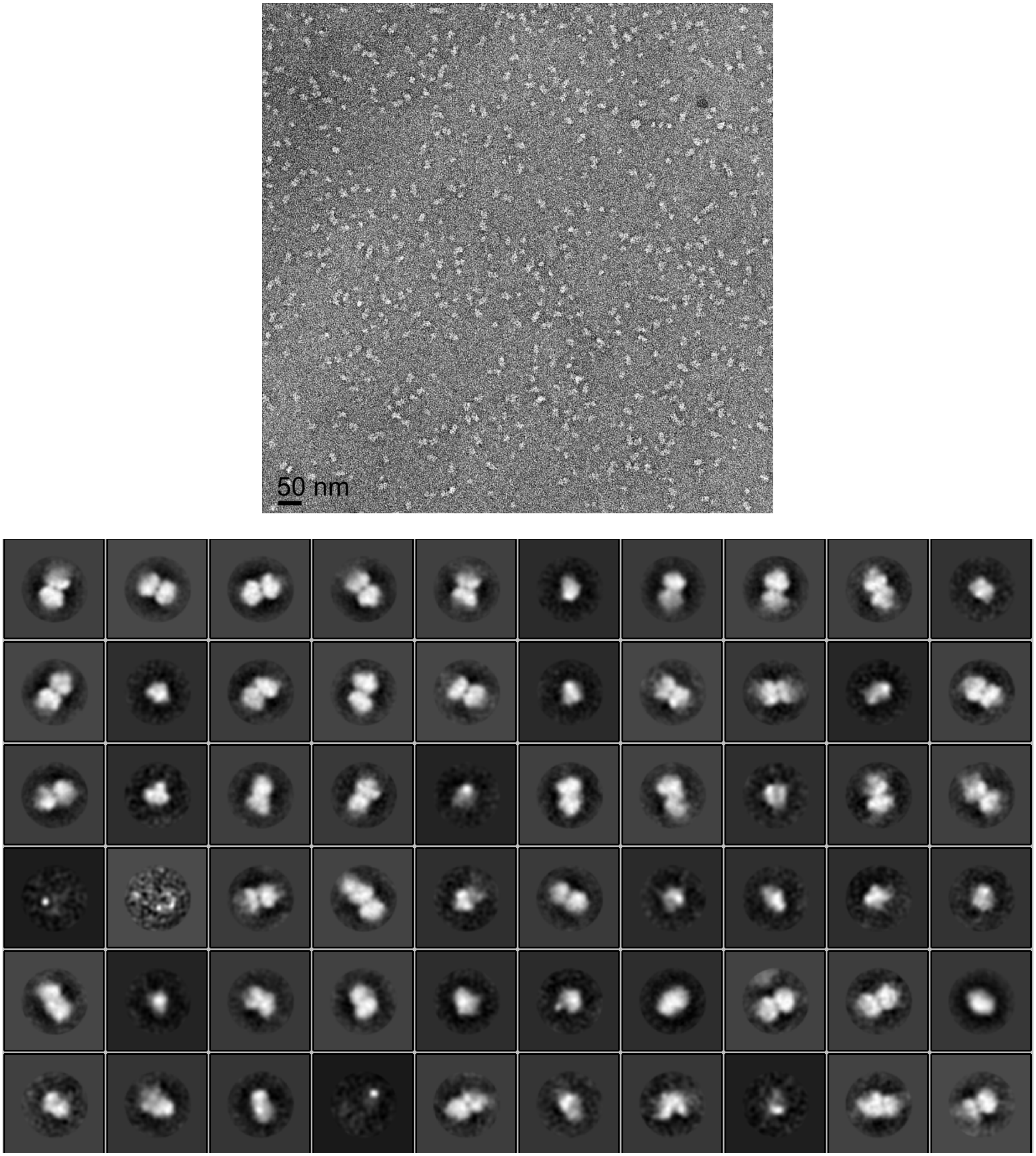

Figure S2: Negative stain grids prepared anaerobically. Representative negative stain micrograph and 60 representative 2Ds of anaerobically prepared grids.

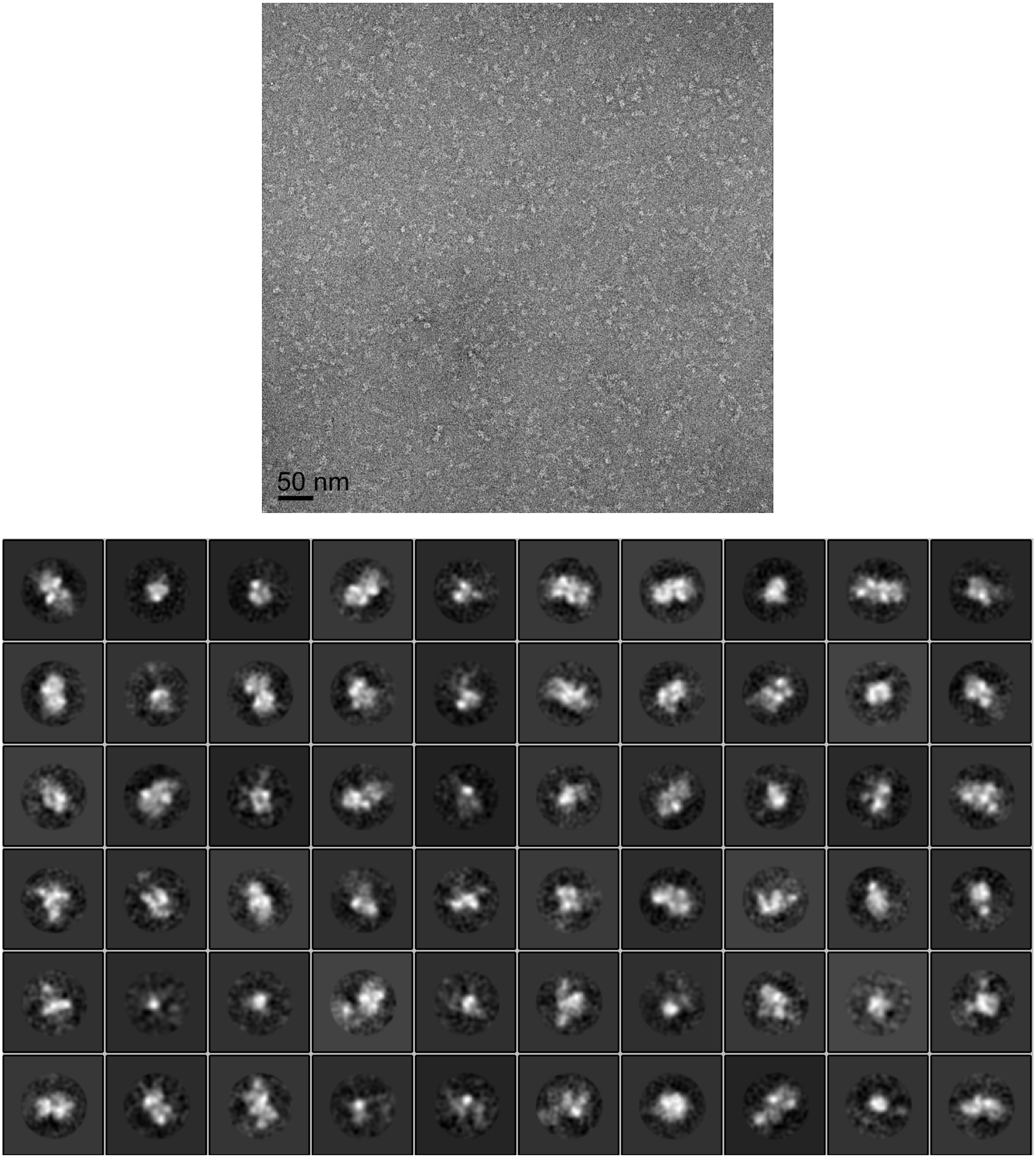

Figure S3: Negative stain grids prepared aerobically. Representative negative stain micrograph and 60 representative 2Ds of aerobically prepared grids.

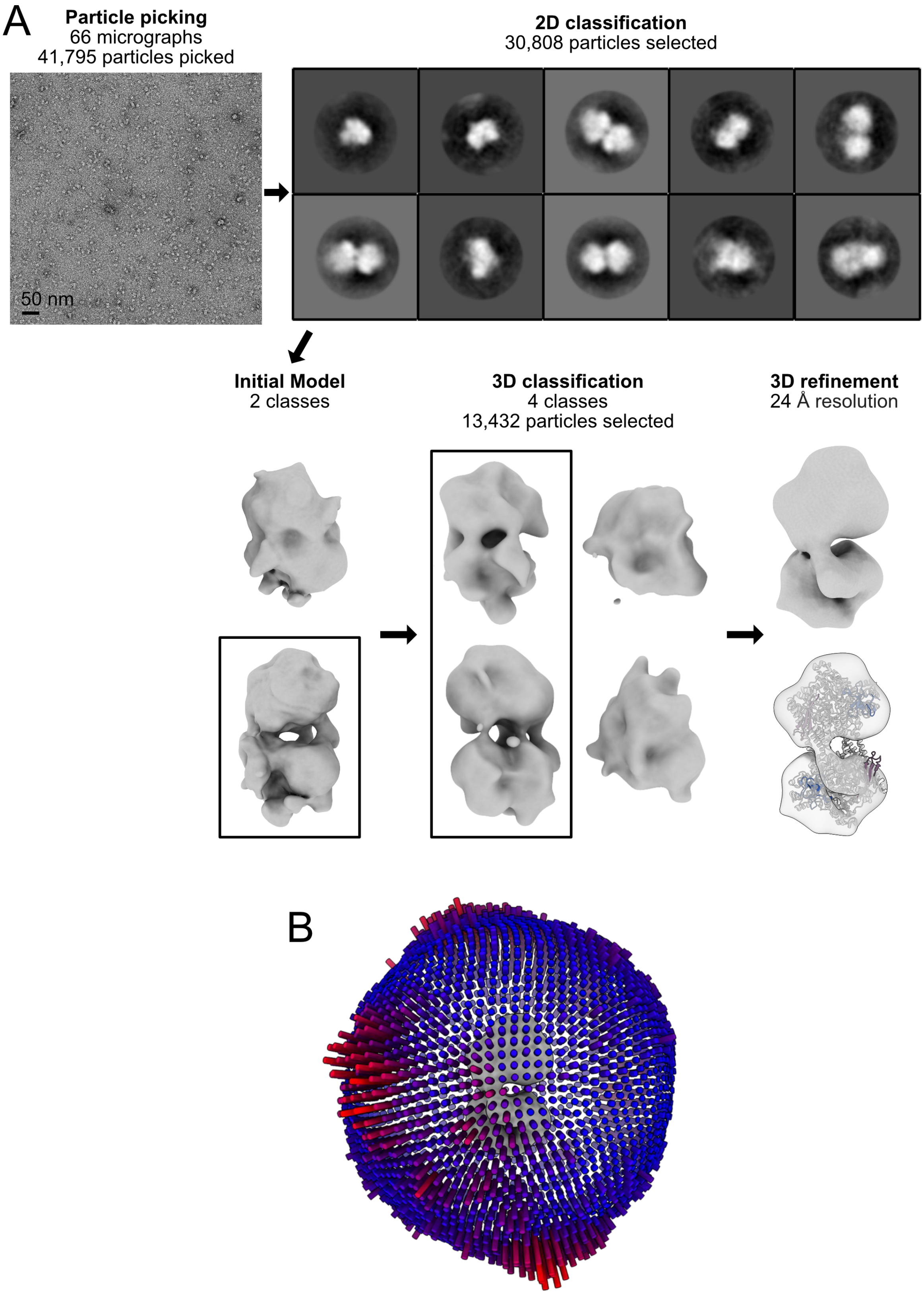

Figure S4: Negative stain processing workflow. A) Scheme describing the data processing steps to generate the negative stain map. BSS($\alpha\beta\gamma$)$_2$ (PDB ID: 5BWE)[15] was fit into the final map. B) Particle orientation distribution for the final negative stain reconstruction.

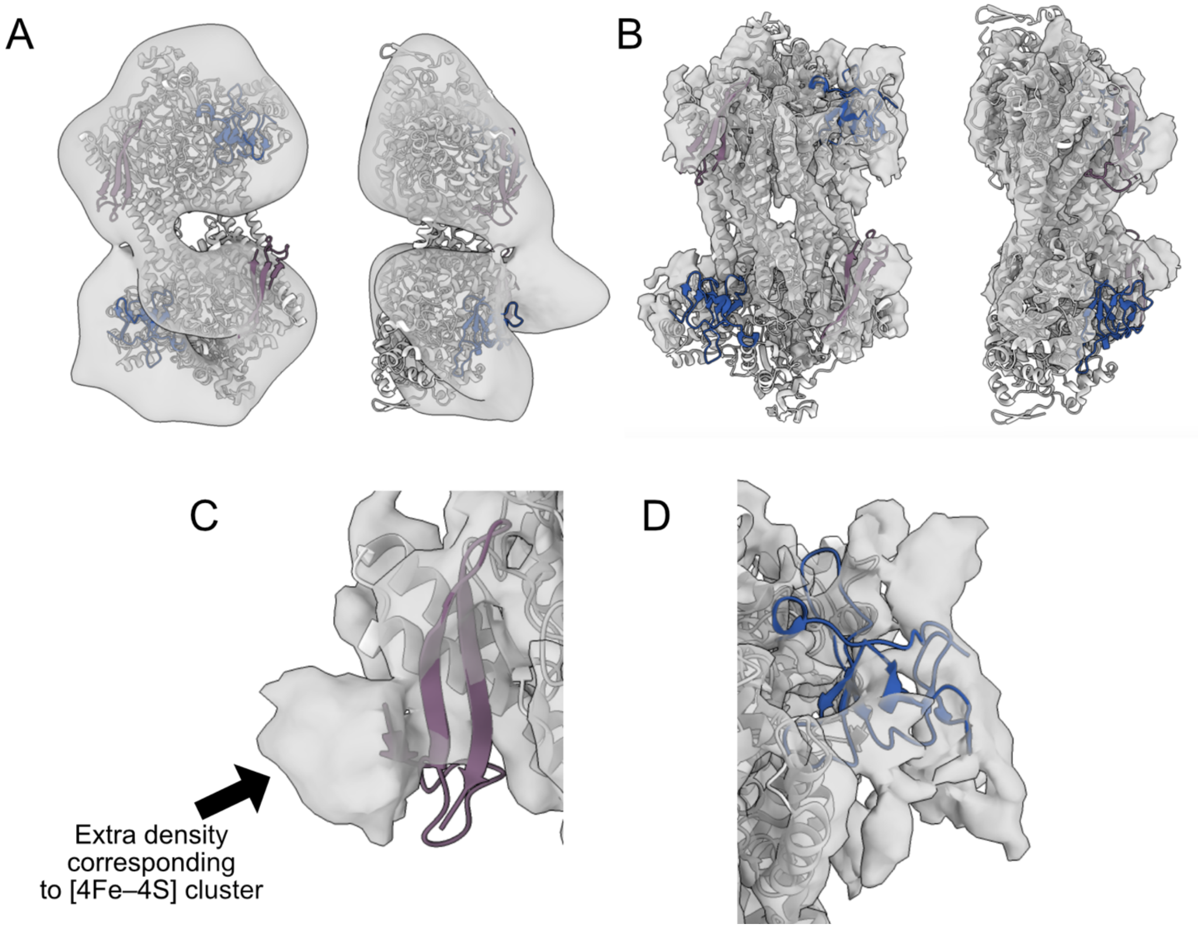

Figure S5: BSS(αβγ)$_2$ (PDB ID: 5BWE)[15] modelled into the negative stain map and low-resolution cryo-EM map. BSSα is shown in grey, BSSγ is shown in purple, and BSSβ is shown in blue. A) When BSS(αβγ)$_2$ is modelled into the negative stain map, it is clear that there are two MASSα subunits. More density is observed for one side of the dimer than the other, perhaps suggesting that different accessory subunits are bound to the different sides of the MASSα dimer. B) When BSS(αβγ)$_2$ is modelled into the low-resolution cryo-EM map, one side of the BSS(αγ)$_2$ fit into the density well, whereas there is no density for the glycyl radical domain and N-terminus of BSSα on the other side of the dimer. This asymmetry in the dimer is consistent with the negative stain map. C) Extra density for the [4Fe–4S] cluster of MASSγ is apparent. D) Extra density in the low-resolution map of MASS was unaccounted for. This density is in the region on α where β binds to BSS; however, BSSβ does not fit well into this density.

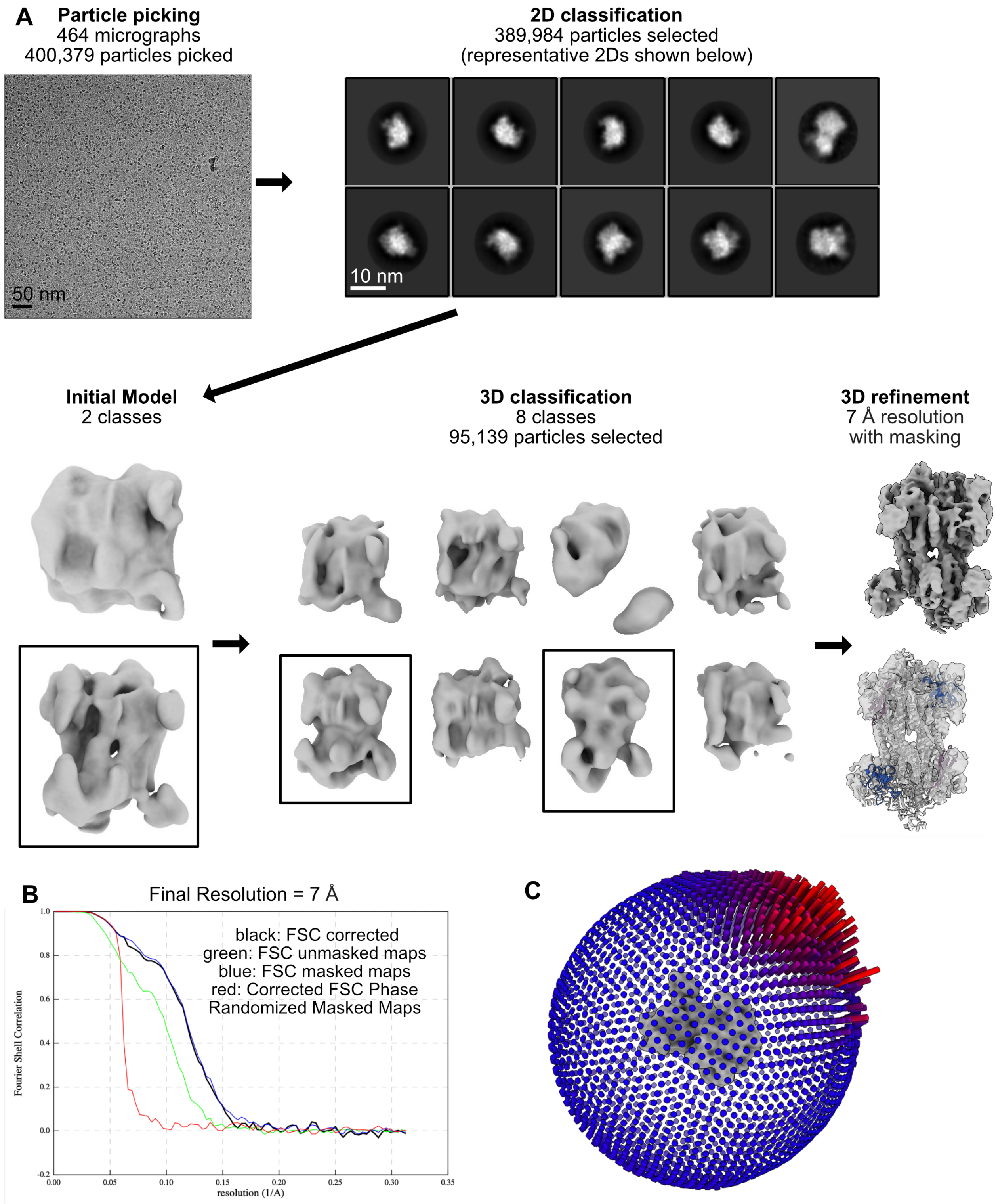

Figure S6: Processing workflow for low-resolution cryo-EM data set. A) Scheme describing the data processing steps to generate the low-resolution cryo-EM map. BSS(αβγ)$_2$ (PDB ID: 5BWE)[15] was fit into the final map. B) Fourier Shell Correlation (FSC) plots for the final low-resolution cryo-EM map. C) Particle orientation distribution for the final low-resolution cryo-EM map.

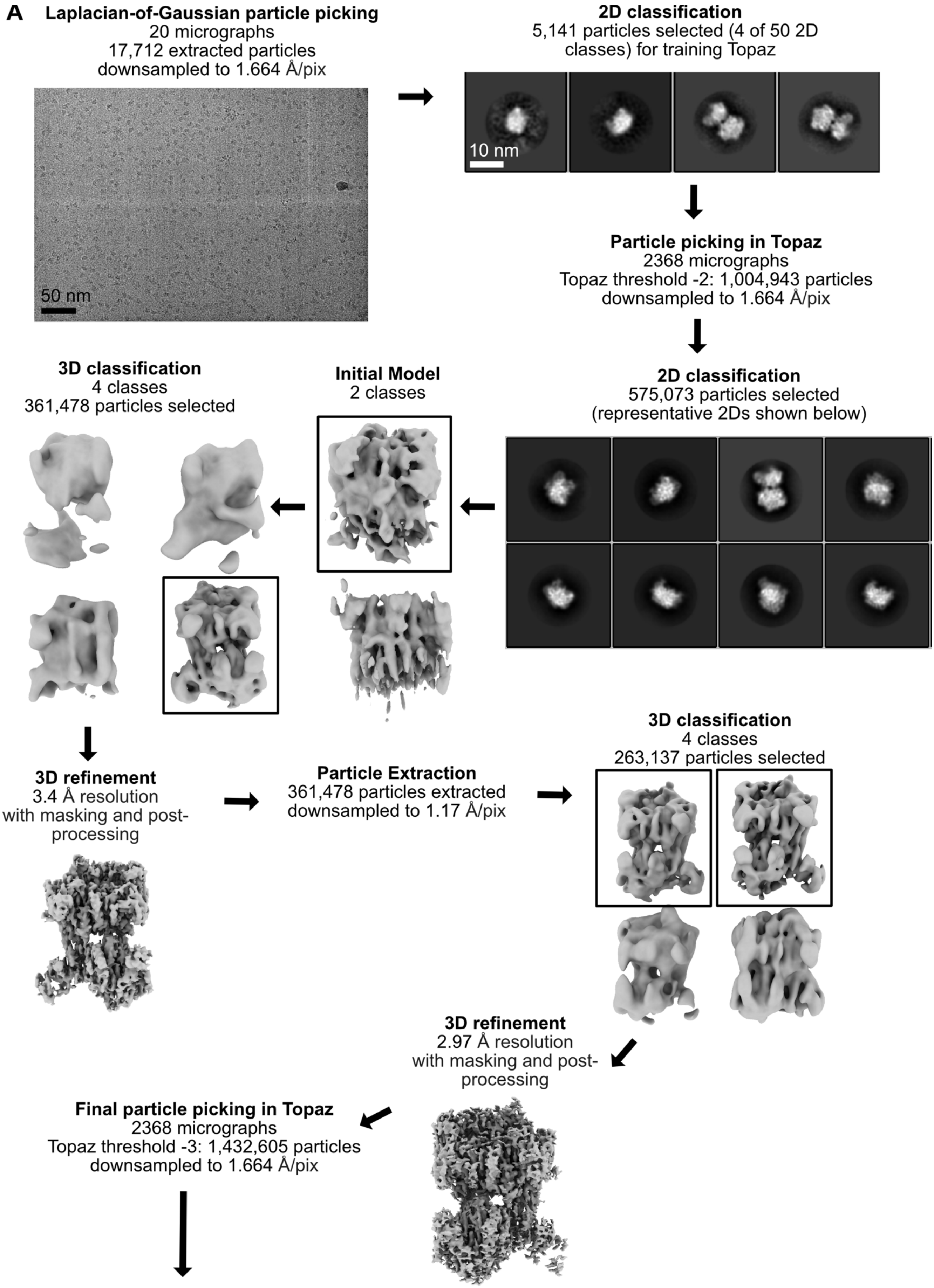

A
Laplacian-of-Gaussian particle picking
20 micrographs
17,712 extracted particles
downsampled to 1.664 Å/pix
50 nm
2D classification
5,141 particles selected (4 of 50 2D classes) for training Topaz
10 nm
Particle picking in Topaz
2368 micrographs
Topaz threshold -2: 1,004,943 particles
downsampled to 1.664 Å/pix
2D classification
575,073 particles selected
(representative 2Ds shown below)
Initial Model
2 classes
3D classification
4 classes
361,478 particles selected
3D refinement
3.4 Å resolution
with masking and post-processing
Particle Extraction
361,478 particles extracted
downsampled to 1.17 Å/pix
3D classification
4 classes
263,137 particles selected
3D refinement
2.97 Å resolution
with masking and post-processing
Final particle picking in Topaz
2368 micrographs
Topaz threshold -3: 1,432,605 particles
downsampled to 1.664 Å/pix

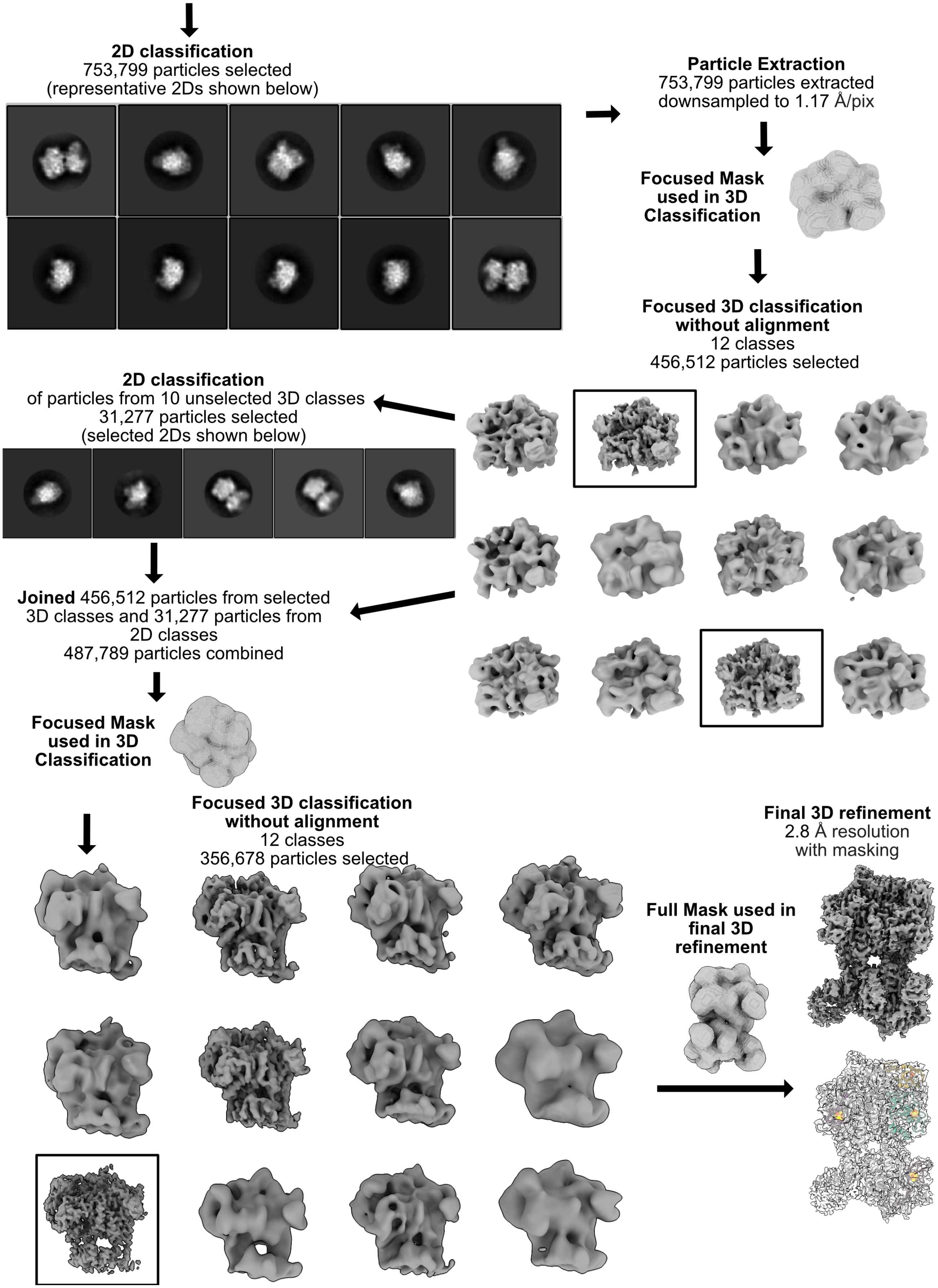
2D classification
753,799 particles selected
(representative 2Ds shown below)
Particle Extraction
753,799 particles extracted
downsampled to 1.17 Å/pix
Focused Mask used in 3D Classification
Focused 3D classification without alignment
12 classes
456,512 particles selected
2D classification
of particles from 10 unselected 3D classes
31,277 particles selected
(selected 2Ds shown below)
Joined 456,512 particles from selected 3D classes and 31,277 particles from 2D classes
487,789 particles combined
Focused Mask used in 3D Classification
Focused 3D classification without alignment
12 classes
356,678 particles selected
Full Mask used in final 3D refinement
Final 3D refinement
2.8 Å resolution
with masking

B

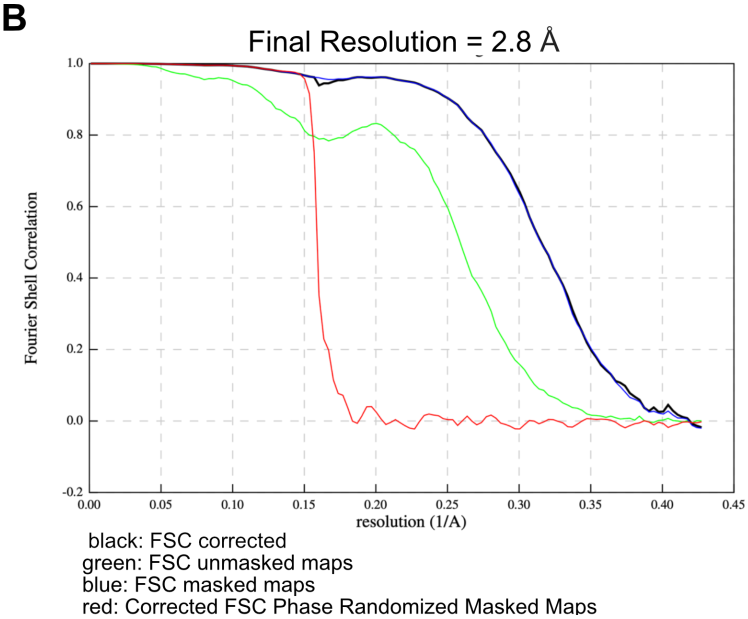

C

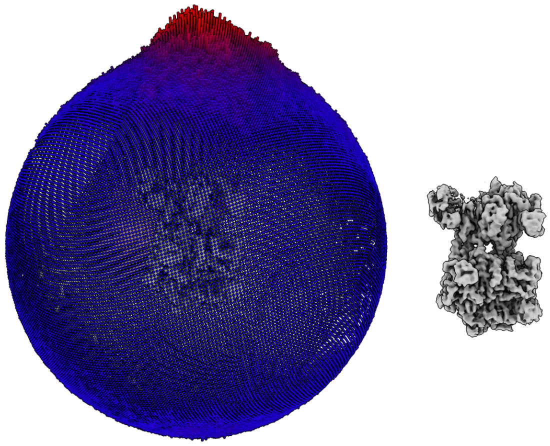

Figure S7: Processing workflow for high-resolution cryo-EM data set. A) Scheme describing the data processing steps to generate the high-resolution cryo-EM map. Workflow continues on 3 pages. B) Fourier Shell Correlation (FSC) plots for the final high-resolution cryo-EM map. C) Particle orientation distribution for the final high-resolution cryo-EM map.

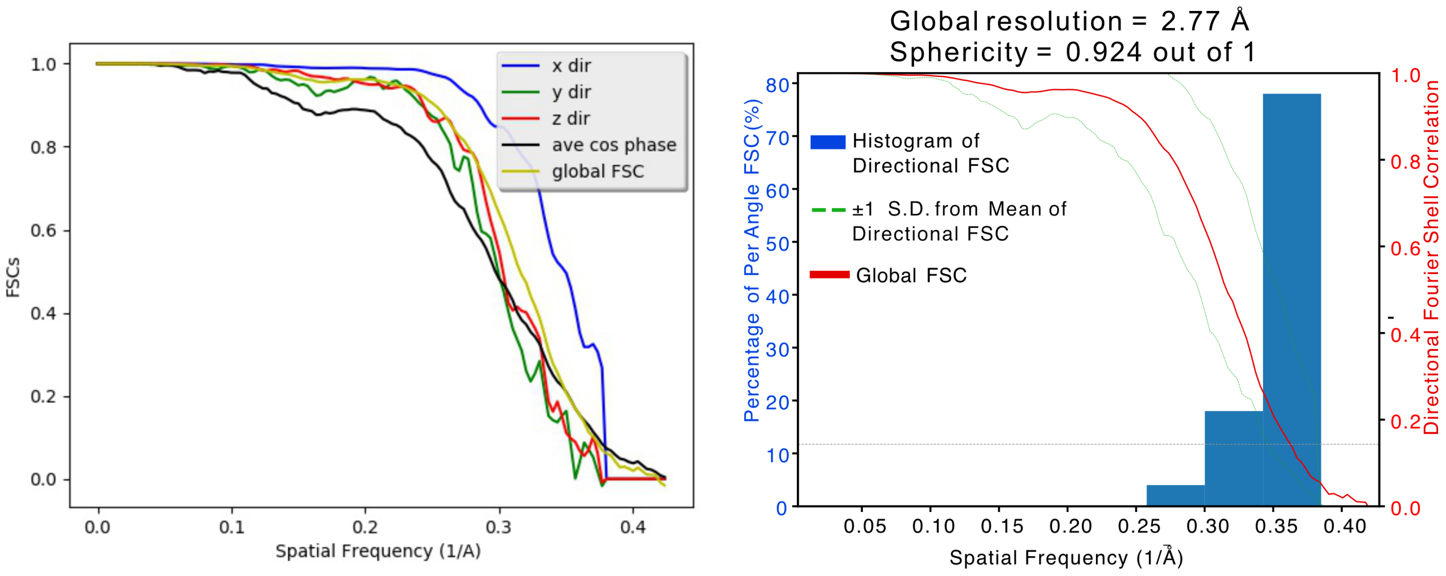

Figure S8: The 3D Fourier Shell Correlation (3DFSC) plots for the final high-resolution cryo-EM map.[16]

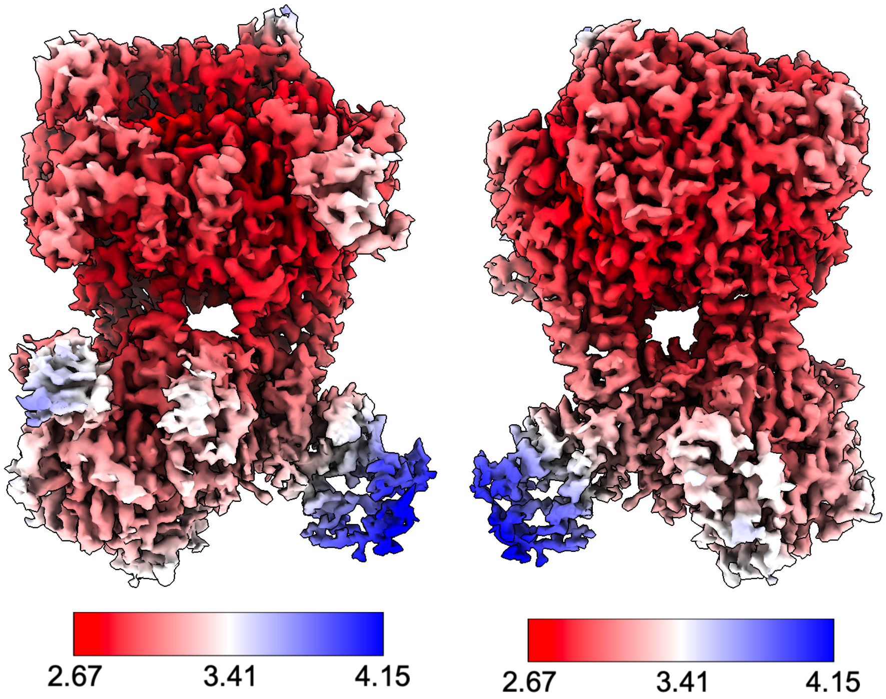

Figure S9: Local resolution maps of the final high-resolution cryo-EM map. Two views rotated 180 ˚ from each other of a local resolution map generated in RELION. Resolution ranged from 2.67 Å in the core of MASSα to 4.14 Å on the periphery of MASSα'.

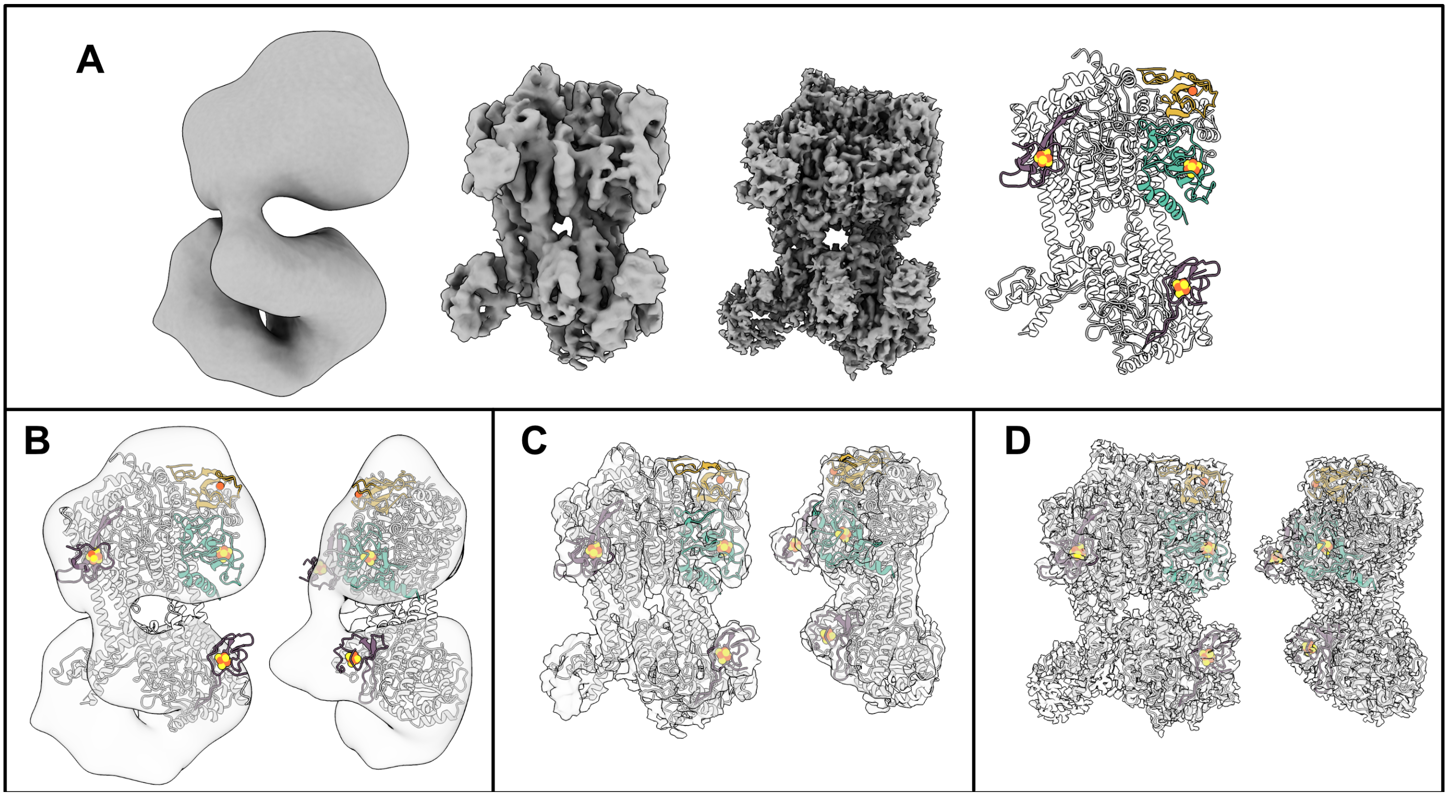

Figure S10: Final model of MASSα$_2$βγ$_2$δ and comparison of negative stain, low-resolution cryo-EM, and high-resolution cryo-EM maps. A) From left to right, the negative stain map, low-resolution cryo-EM map, high-resolution cryo-EM map, and final MASSα$_2$βγ$_2$δ model are shown. B-D) The MASSα$_2$βγ$_2$δ complex docked into the negative stain map (B), low-resolution cryo-EM map (C), high-resolution cryo-EM map (D). Two views rotated 90 ˚ are shown for each map.

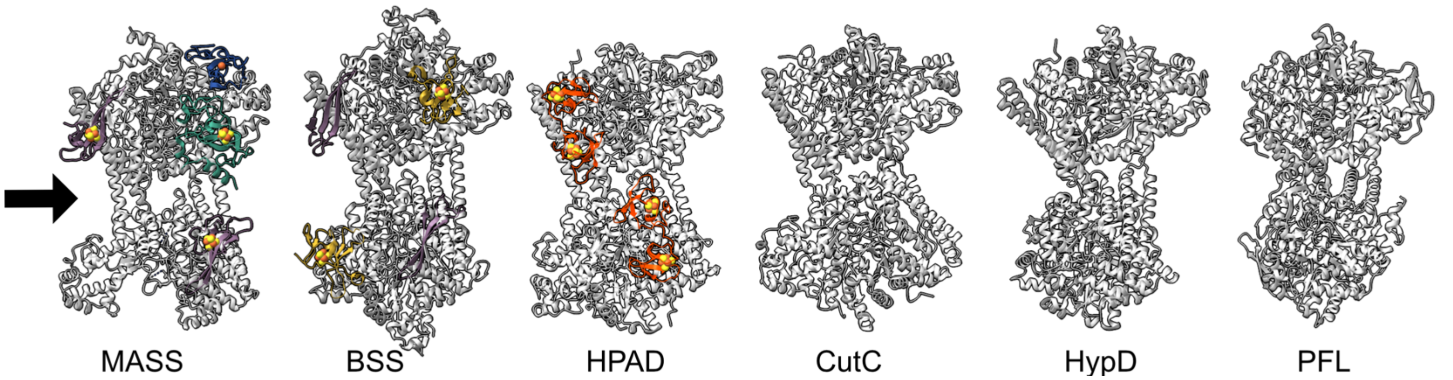

Figure S11: Dimer interfaces are conserved among GREs. The MASS α/α' dimer interface, denoted with an arrow, is similar to other GREs across the superfamily, including benzylsuccinate synthase (BSS, PDB ID 5BWE[15]), 4hydroxyphenylacetate decarboxylase (HPAD, PDB ID 2Y8N[17]), choline trimethylamine-lyase (CutC, PDB ID 5FAU[18]), hydroxyproline dehydratase (HypD, PDB ID 6VXC[19]), pyruvate formate-lyase (PFL, PDB ID 1H18[20]). CutC, HypD, and PFL consist of a dimer of catalytic subunits, with no accessory subunits. HPAD binds an accessory subunit (HPADγ = red); BSS binds two accessory subunits (BSSβ = yellow, BSSγ = purple); and MASS binds three accessory subunits (MASSβ = green, MASSγ = purple, MASSδ = blue).

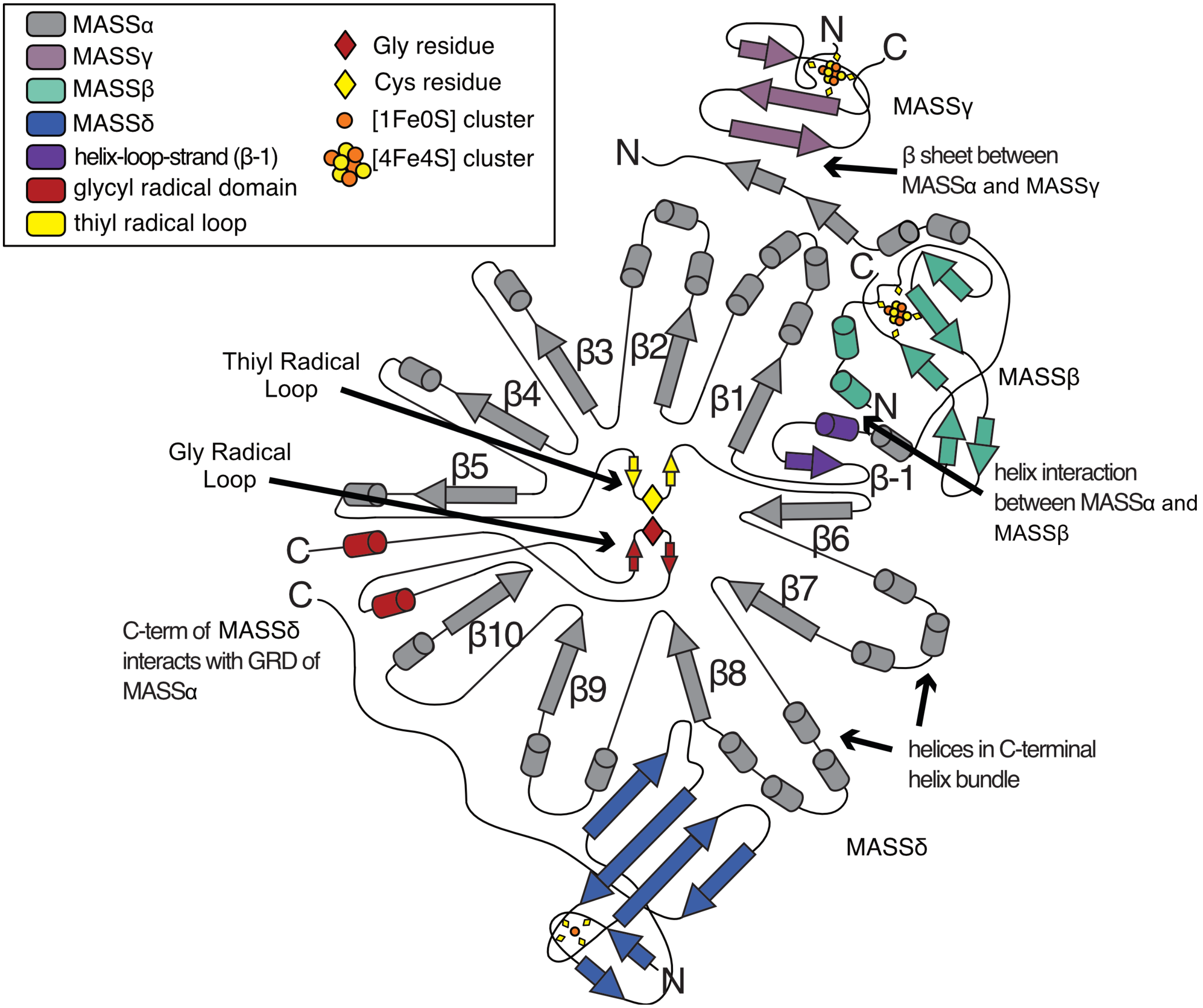

Figure S12: Topology diagram for MASS complex.[21]

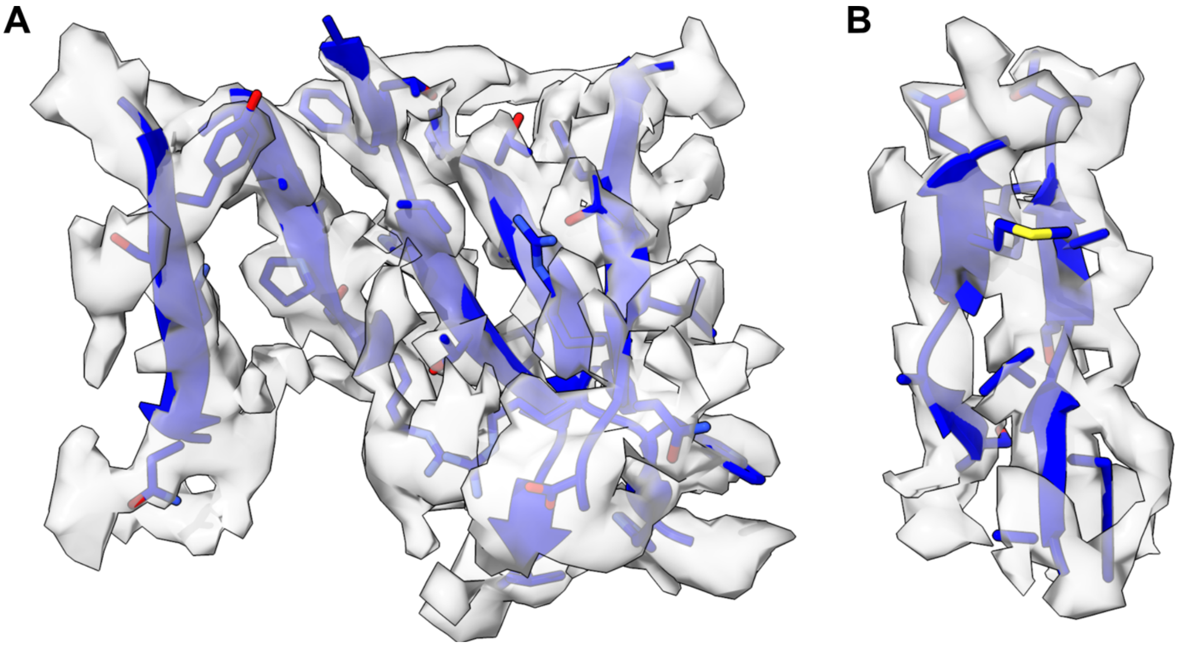

Figure S13: Density for the β-strands of the β/α-half barrels. A) Density for the first 5-stranded β/α-half barrel of MASSα. B) Density for β6 and β7 of the second 5-stranded β/α-half barrel of MASSα'.

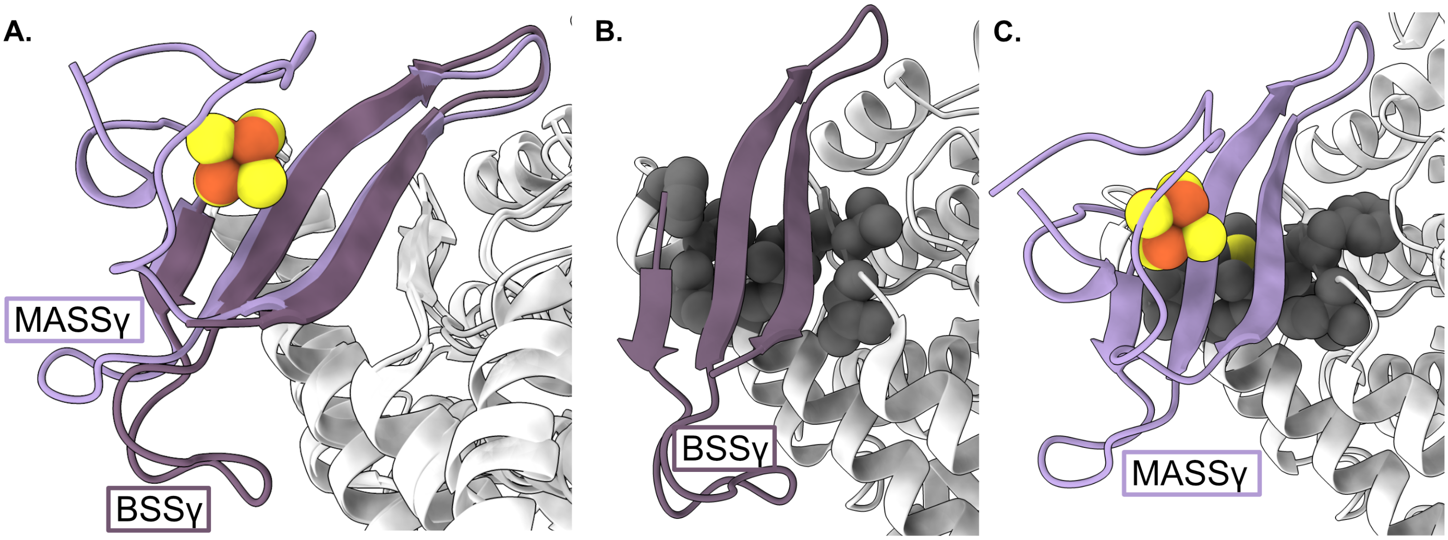

Figure S14: Comparison of BSSγ and MASSγ. A) β-strands of BSSγ (PDB ID: 5BWE[15]) overlay well with MASSγ; however, the N-terminus, C-terminus and 4Fe–4S cluster of MASSγ could be built into the high-resolution cryo-EM map. B) BSSγ covers a hydrophobic patch (dark grey) on BSSα. C) Similarly, MASSγ covers a hydrophobic patch (dark grey) on MASSα.

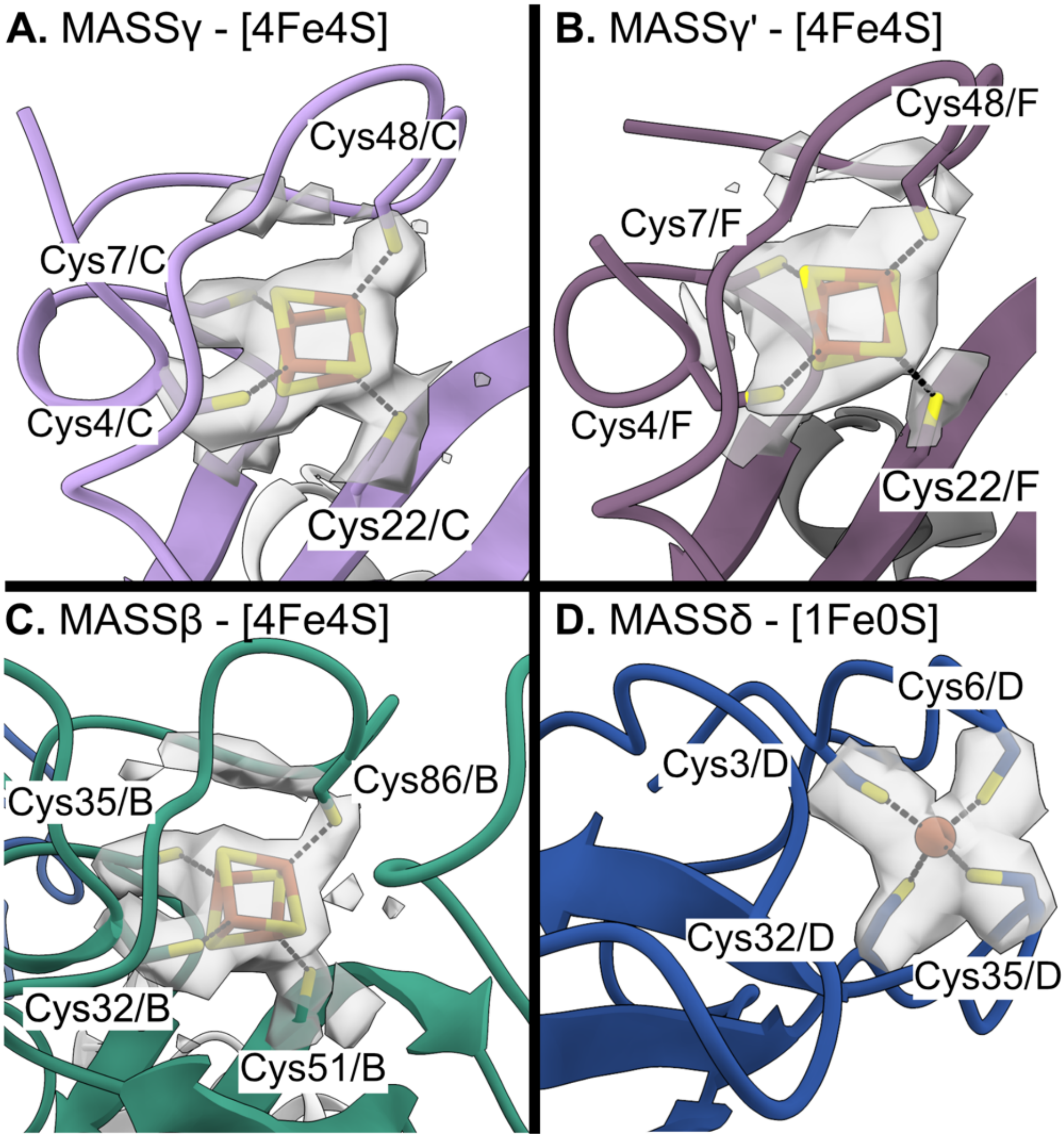

Figure S15: Density for FeS clusters in accessory subunits. A) A 4Fe-4S cluster is ligated by 4 Cys residues (C4, C7, C22, and C48) in MASSγ. B) A similar 4Fe-4S cluster is ligated by 4 Cys residues (C4, C7, C22, and C48) in MASSγ'. C) A 4Fe-4S cluster is ligated by 4 Cys residues (C32, C35, C51, and C86) in MASSβ. D) Unlike other GRE FeS-containing subunits, which all coordinate a 4Fe–4S cluster, MASSδ contains a single Fe ion ligated by 4 Cys residues (C3, C6, C32, and C35).

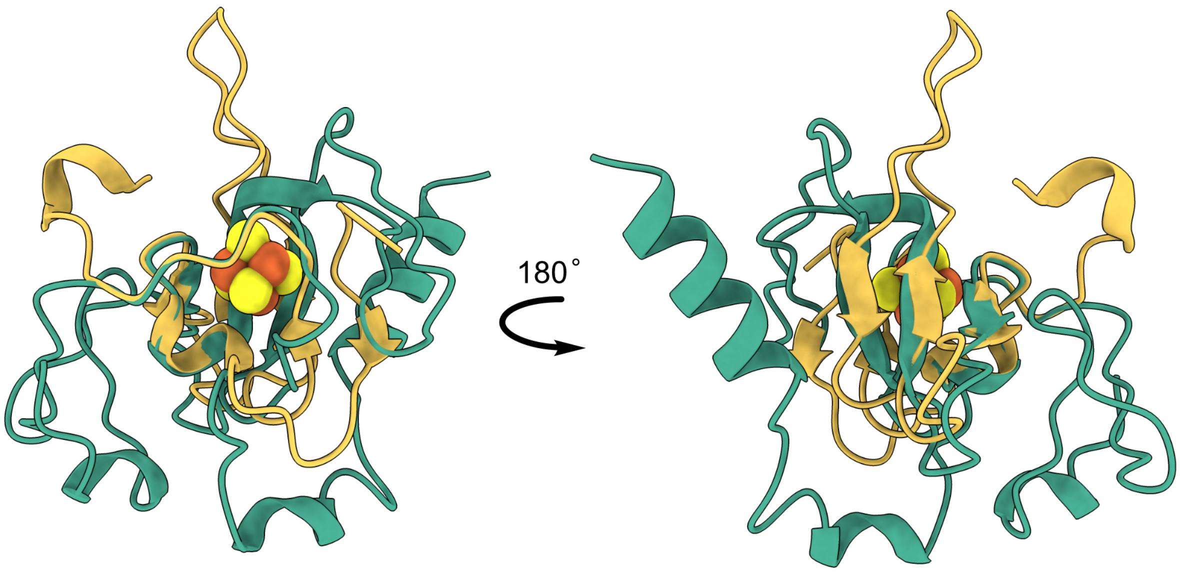

Figure S16: Overlay of MASSβ and BSSβ. MASSβ (green, PDB ID: 5BWE) and BSSβ (yellow) adopt a fold resembling high potential iron-sulfur proteins. A structural overlay of BSSβ and HPADγ is presented in Funk et al.[22]

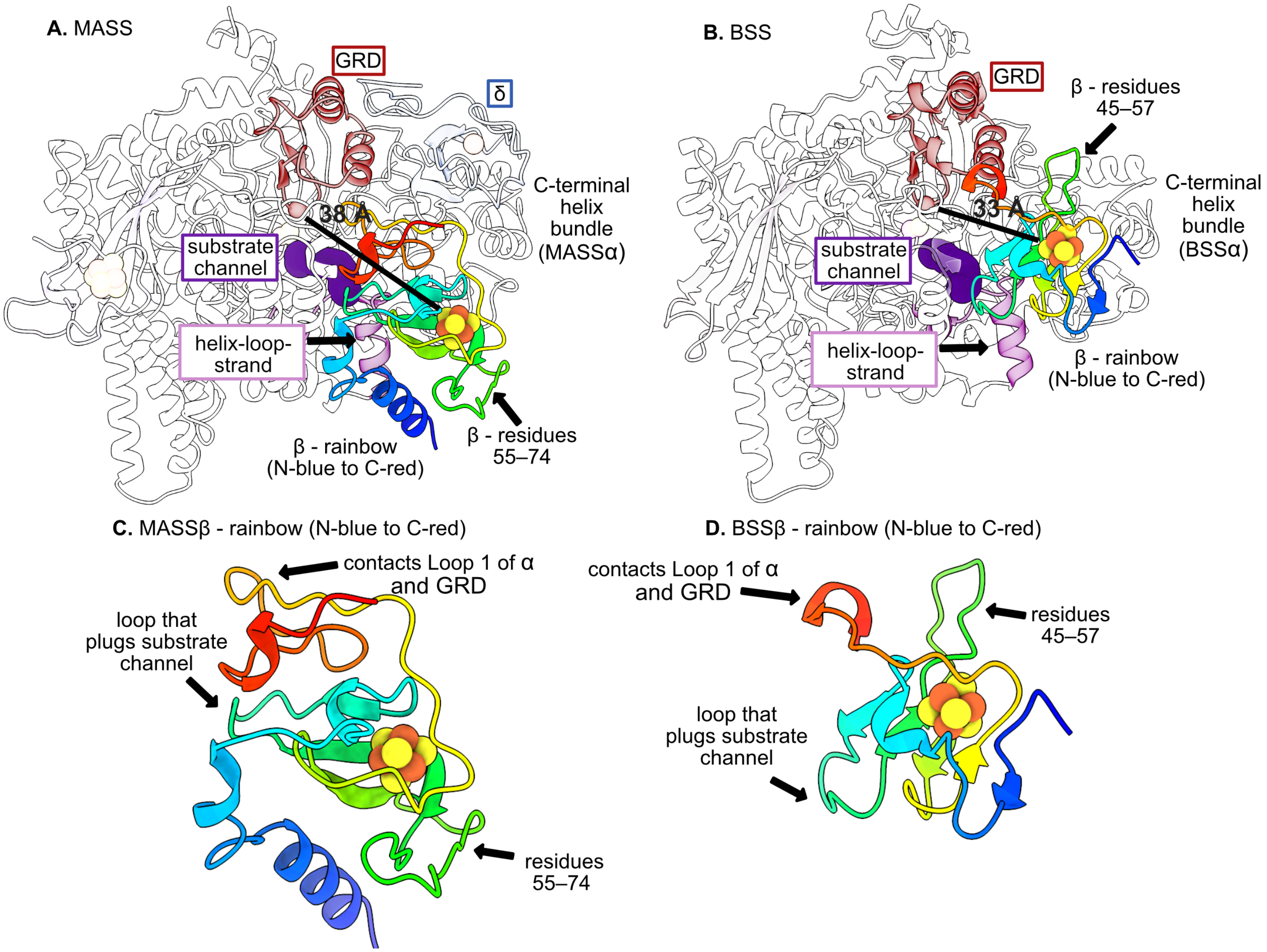

Figure S17: Comparison of the location and structure of β subunits of BSS and MASS. MASSβ and BSSβ (PDB ID: 5BWE[15]) are colored as rainbow, with red corresponding to the C-terminus and blue corresponding to the N-terminus. The 4Fe–4S clusters in the β subunits are shown as spheres. A-B) MASSβ binds to MASSα in a similar position as BSSβ binds to BSSα; however, the core of the β subunits that bind the 4Fe–4S clusters are rotated approximately 90 ° relative to one another. C-D) Panels A-B are simplified to only show MASSβ (C) and BSSβ (D).

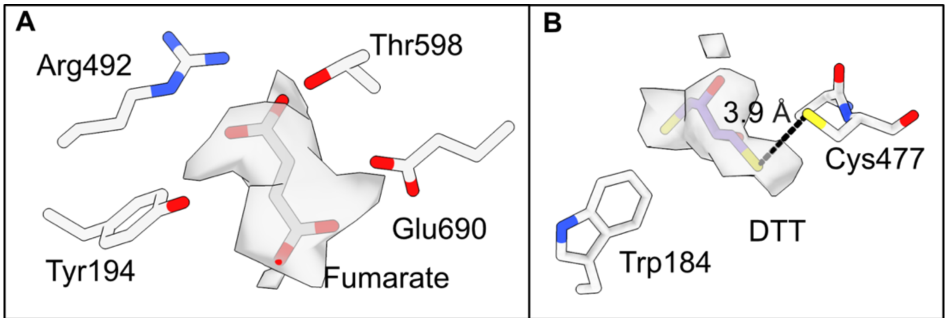

Figure S18: Density for modelled substrates in final MASS structure. A) Fumarate was added to all purification buffers and was observed in the active site of MASSα. Residues capable of H-bonding to fumarate are shown as sticks. B) No alkane substrate was added to buffers, but density was observed in the presumed alkane binding site. Upon analysis of buffer conditions, dithiothreitol (DTT) gave the best fit and was thus modelled into the density. Cys477 (the catalytic Cys) and Trp184 (proposed to gate the hydrocarbon substrate channel) are shown as sticks.

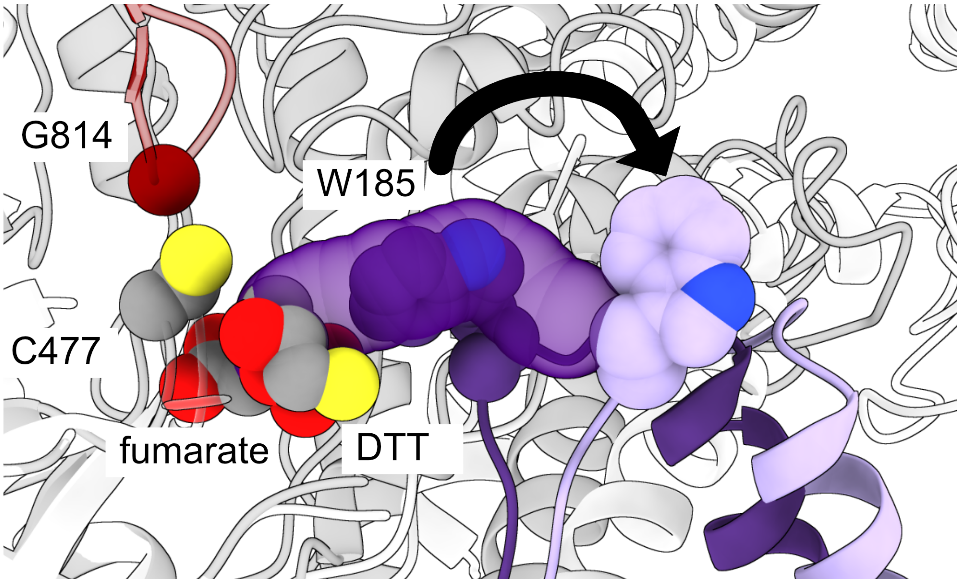

Figure S19: Trp185 is proposed to gate substrate entry. MASSα corresponds to darker colors and MASSα' corresponds to lighter colors. Trp185, shown as spheres, is positioned towards the active site in MASSα (dark purple) and away from the active site in MASSα' (light purple). DTT, fumarate, and Cys477 are shown in grey and the Gly loop is shown in dark red.

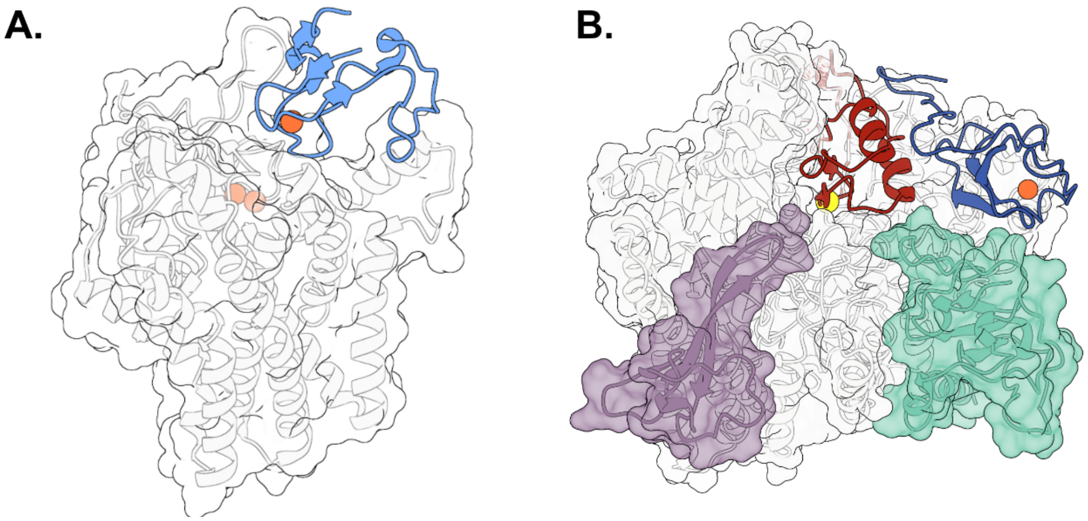

Figure S20: Distinct orientations of the rubredoxin-like domain binding in AlkB-AlkG and MASSα-MASSδ complexes. Fe ions are shown as orange spheres. AlkB binds its rubredoxin-like partner protein, AlkG (light blue) (A), in a different orientation than MASSα binds MASSδ (blue) (B). In AlkB, the diiron center is thought to be reduced via the Fe ion of the rubredoxin-like domain in AlkG. (PDB ID: 8F6T[23], 13.6 Å from the Fe ion in AlkG to the closest Fe ion in AlkB). In MASS, the rubredoxin-like domain faces away from the MASSα active site (essential glycine residue is shown as a yellow sphere, 39.4 Å from the Fe ion in MASSδ to the Cα of the essential glycine).

References:

(1) Scheres, S. H. W. RELION: Implementation of a Bayesian Approach to Cryo-EM Structure Determination. *Journal of Structural Biology* **2012**, *180* (3), 519–530. https://doi.org/10.1016/j.jsb.2012.09.006.

(2) Wagner, T.; Merino, F.; Stabrin, M.; Moriya, T.; Antoni, C.; Apelbaum, A.; Hagel, P.; Sitsel, O.; Raisch, T.; Prumbaum, D.; Quentin, D.; Roderer, D.; Tacke, S.; Siebolds, B.; Schubert, E.; Shaikh, T. R.; Lill, P.; Gatsogiannis, C.; Raunser, S. SPHIRE-crYOLO Is a Fast and Accurate Fully Automated Particle Picker for Cryo-EM. *Commun Biol* **2019**, *2* (1), 218. https://doi.org/10.1038/s42003-019-0437-z.

(3) Moriya, T.; Saur, M.; Stabrin, M.; Merino, F.; Voicu, H.; Huang, Z.; Penczek, P. A.; Raunser, S.; Gatsogiannis, C. High-Resolution Single Particle Analysis from Electron Cryo-Microscopy Images Using SPHIRE. *JoVE* **2017**, No. 123, 55448. https://doi.org/10.3791/55448.

(4) Bepler, T.; Morin, A.; Rapp, M.; Brasch, J.; Shapiro, L.; Noble, A. J.; Berger, B. Positive-Unlabeled Convolutional Neural Networks for Particle Picking in Cryo-Electron Micrographs. *Nat Methods* **2019**, *16* (11), 1153–1160. https://doi.org/10.1038/s41592-019-0575-8.

(5) Jumper, J.; Evans, R.; Pritzel, A.; Green, T.; Figurnov, M.; Ronneberger, O.; Tunyasuvunakool, K.; Bates, R.; Žídek, A.; Potapenko, A.; Bridgland, A.; Meyer, C.; Kohl, S. A. A.; Ballard, A. J.; Cowie, A.; Romera-Paredes, B.; Nikolov, S.; Jain, R.; Adler, J.; Back, T.; Petersen, S.; Reiman, D.; Clancy, E.; Zielinski, M.; Steinegger, M.; Pacholska, M.; Berghammer, T.; Bodenstein, S.; Silver, D.; Vinyals, O.; Senior, A. W.; Kavukcuoglu, K.; Kohli, P.; Hassabis, D. Highly Accurate Protein Structure Prediction with AlphaFold. *Nature* **2021**, *596* (7873), 583–589. https://doi.org/10.1038/s41586-021-03819-2.

(6) Goddard, T. D.; Huang, C. C.; Meng, E. C.; Pettersen, E. F.; Couch, G. S.; Morris, J. H.; Ferrin, T. E. UCSF ChimeraX: Meeting Modern Challenges in Visualization and Analysis. *Protein Science* **2018**, *27* (1), 14–25. https://doi.org/10.1002/pro.3235.

(7) Emsley, P.; Lohkamp, B.; Scott, W. G.; Cowtan, K. Features and Development of *Coot*. *Acta Crystallogr D Biol Crystallogr* **2010**, *66* (4), 486–501. https://doi.org/10.1107/S0907444910007493.

(8) Adams, P. D.; Afonine, P. V.; Bunkóczi, G.; Chen, V. B.; Davis, I. W.; Echols, N.; Headd, J. J.; Hung, L.-W.; Kapral, G. J.; Grosse-Kunstleve, R. W.; McCoy, A. J.; Moriarty, N. W.; Oeffner, R.; Read, R. J.; Richardson, D. C.; Richardson, J. S.; Terwilliger, T. C.; Zwart, P. H. *PHENIX* : A Comprehensive Python-Based System for Macromolecular Structure Solution. *Acta Crystallogr D Biol Crystallogr* **2010**, *66* (2), 213–221. https://doi.org/10.1107/S0907444909052925.

(9) Williams, C. J.; Headd, J. J.; Moriarty, N. W.; Prisant, M. G.; Videau, L. L.; Deis, L. N.; Verma, V.; Keedy, D. A.; Hintze, B. J.; Chen, V. B.; Jain, S.; Lewis, S. M.; Arendall, W. B.; Snoeyink, J.; Adams, P. D.; Lovell, S. C.; Richardson, J. S.; Richardson, D. C. MolProbity: More and Better Reference Data for Improved All-atom Structure Validation. *Protein Science* **2018**, *27* (1), 293–315. https://doi.org/10.1002/pro.3330.

(10) Barad, B. A.; Echols, N.; Wang, R. Y.-R.; Cheng, Y.; DiMaio, F.; Adams, P. D.; Fraser, J. S. EMRinger: Side Chain–Directed Model and Map Validation for 3D Cryo-Electron Microscopy. *Nat Methods* **2015**, *12* (10), 943–946. https://doi.org/10.1038/nmeth.3541.

(11) Peng, Y.; Veneziano, S. E.; Gillispie, G. D.; Broderick, J. B. Pyruvate Formate-Lyase, Evidence for an Open Conformation Favored in the Presence of Its Activating Enzyme. *Journal of Biological Chemistry* **2010**, *285* (35), 27224–27231. https://doi.org/10.1074/jbc.M109.096875.

(12) Vey, J. L.; Yang, J.; Li, M.; Broderick, W. E.; Broderick, J. B.; Drennan, C. L. Structural Basis for Glycyl Radical Formation by Pyruvate Formate-Lyase Activating Enzyme. *Proc. Natl. Acad. Sci. U.S.A.* **2008**, *105* (42), 16137–16141. https://doi.org/10.1073/pnas.0806640105.

(13) Mastronarde, D. N. Automated Electron Microscope Tomography Using Robust Prediction of Specimen Movements. *Journal of Structural Biology* **2005**, *152* (1), 36–51. https://doi.org/10.1016/j.jsb.2005.07.007.

(14) Liebschner, D.; Afonine, P. V.; Baker, M. L.; Bunkóczi, G.; Chen, V. B.; Croll, T. I.; Hintze, B.; Hung, L.-W.; Jain, S.; McCoy, A. J.; Moriarty, N. W.; Oeffner, R. D.; Poon, B. K.; Prisant, M. G.; Read, R. J.; Richardson, J. S.; Richardson, D. C.; Sammito, M. D.; Sobolev, O. V.; Stockwell, D. H.; Terwilliger, T. C.; Urzhumtsev, A. G.; Videau, L. L.; Williams, C. J.; Adams, P. D. Macromolecular Structure Determination Using X-Rays, Neutrons and Electrons: Recent

Developments in *Phenix*. *Acta Crystallogr D Struct Biol* **2019**, *75* (10), 861–877. https://doi.org/10.1107/S2059798319011471.
(15) Funk, M. A.; Marsh, E. N. G.; Drennan, C. L. Substrate-Bound Structures of Benzylsuccinate Synthase Reveal How Toluene Is Activated in Anaerobic Hydrocarbon Degradation. *Journal of Biological Chemistry* **2015**, *290* (37), 22398–22408. https://doi.org/10.1074/jbc.M115.670737.
(16) Tan, Y. Z.; Baldwin, P. R.; Davis, J. H.; Williamson, J. R.; Potter, C. S.; Carragher, B.; Lyumkis, D. Addressing Preferred Specimen Orientation in Single-Particle Cryo-EM through Tilting. *Nat Methods* **2017**, *14* (8), 793–796. https://doi.org/10.1038/nmeth.4347.
(17) Martins, B. M.; Blaser, M.; Feliks, M.; Ullmann, G. M.; Buckel, W.; Selmer, T. Structural Basis for a Kolbe-Type Decarboxylation Catalyzed by a Glycyl Radical Enzyme. *J. Am. Chem. Soc.* **2011**, *133* (37), 14666–14674. https://doi.org/10.1021/ja203344x.
(18) Bodea, S.; Funk, M. A.; Balskus, E. P.; Drennan, C. L. Molecular Basis of C–N Bond Cleavage by the Glycyl Radical Enzyme Choline Trimethylamine-Lyase. *Cell Chemical Biology* **2016**, *23* (10), 1206–1216. https://doi.org/10.1016/j.chembiol.2016.07.020.
(19) Backman, L. R.; Huang, Y. Y.; Andorfer, M. C.; Gold, B.; Raines, R. T.; Balskus, E. P.; Drennan, C. L. Molecular Basis for Catabolism of the Abundant Metabolite Trans-4-Hydroxy-L-Proline by a Microbial Glycyl Radical Enzyme. *eLife* **2020**, *9*, e51420. https://doi.org/10.7554/eLife.51420.
(20) Becker, A.; Kabsch, W. X-Ray Structure of Pyruvate Formate-Lyase in Complex with Pyruvate and CoA. *Journal of Biological Chemistry* **2002**, *277* (42), 40036–40042. https://doi.org/10.1074/jbc.M205821200.
(21) Bowman, S. E. J.; Backman, L. R. F.; Bjork, R. E.; Andorfer, M. C.; Yori, S.; Caruso, A.; Stultz, C. M.; Drennan, C. L. Solution Structure and Biochemical Characterization of a Spare Part Protein That Restores Activity to an Oxygen-Damaged Glycyl Radical Enzyme. *J Biol Inorg Chem* **2019**, *24* (6), 817–829. https://doi.org/10.1007/s00775-019-01681-2.
(22) Funk, M. A.; Judd, E. T.; Marsh, E. N. G.; Elliott, S. J.; Drennan, C. L. Structures of Benzylsuccinate Synthase Elucidate Roles of Accessory Subunits in Glycyl Radical Enzyme Activation and Activity. *Proc. Natl. Acad. Sci. U.S.A.* **2014**, *111* (28), 10161–10166. https://doi.org/10.1073/pnas.1405983111.
(23) Chai, J.; Guo, G.; McSweeney, S. M.; Shanklin, J.; Liu, Q. Structural Basis for Enzymatic Terminal C–H Bond Functionalization of Alkanes. *Nat Struct Mol Biol* **2023**, *30* (4), 521–526. https://doi.org/10.1038/s41594-023-00958-0.
